# Impacts of climate change on land suitability of key crops in New Zealand

**DOI:** 10.1038/s41598-026-49178-8

**Published:** 2026-04-22

**Authors:** Baptiste Hamon, Hervé Quénol, Clémence Vannier, Thomas A. Cochrane

**Affiliations:** 1 Department of Civil and Environmental Engineering, University of Canterbury, Private Bag 4800, Christchurch, 8140 New Zealand; 2 IRL2046 CliMoA, CNRS-MWLR-BSI, 76 Gerald Street, Lincoln, 7608 New Zealand; 3 Bioeconomy Science Institute, Manaaki Whenua – Landcare Research Group, Lincoln, New Zealand

**Keywords:** Agricultural Land Suitability, Crop Suitability Modelling, Climate Change Adaptation, Climate Variability and Uncertainty, Irrigation Demand, New Zealand Agriculture, Agriculture, Climate sciences, Ecology, Ecology, Environmental sciences, Environmental social sciences, Plant sciences

## Abstract

Climate change threatens global agriculture, food security and nutrition. Understanding its regional impacts is necessary to help agricultural sectors adapt and increase their resilience in these changing climate conditions. In New Zealand (NZ), studies on the impacts of climate change on agriculture have been conducted on a limited number of crops, using different methods and without assessing uncertainties. This study aims to bridge these gaps by applying a consistent method of Land Suitability Analysis (LSA) to four key crops for NZ agriculture – apple, cherry, maize and wheat – and assessing suitability robustness. The results show that historical suitability patterns are consistent with current production areas. An increase in suitability is projected for all the crops in almost all of the South Island of NZ, whereas in the North Island, some of the crops are less suitable, highlighting both constraints and opportunities for future NZ agriculture. The robustness of results varies depending on the climate scenario and period considered. In addition, an increase in net irrigation requirements is also projected for the crops, requiring critical management of future water supplies. The limits of the study include the climate data resolution and the omission of the water availability seasonality and other biotic factors, such as diseases. The framework and methodology developed and applied in this study to New Zealand can readily be adapted for use in other regions.

**Supplementary Information:**

The online version contains supplementary material available at 10.1038/s41598-026-49178-8.

## Introduction

Climate change can have a negative or positive impact on agriculture^1^. It can exacerbate global food security and nutrition by negatively impacting yields and the nutritive value of major crops^1–4^, and exposing livestock and workers to more heat stress^1,5^. By changing environmental conditions, climate change also alters the distribution of crops, the timing of phenological stages and the growing area suitability^1^, where the highly suitable areas for agriculture are projected to decrease worldwide^6^. On the other hand, changing climate conditions may create opportunities by extending suitable areas for agriculture beyond currently cultivated regions, especially in high latitudes or by making land currently used for agriculture suitable for new crops^3,4,6^.

In the last decades, agricultural productivity has been enhanced mainly through the expansion of land and the use of natural resources^7^. However, solutions used in the past are no longer relevant as 91% of all suitable land is already occupied by agriculture, limiting possible expansion^6^, and the need to rely less on the use of finite natural resources and inputs^7,8^. Thus, agricultural systems must evolve and find sustainable options while maintaining profitability within a climate change context. Land Suitability Analysis (LSA), referring to a framework used for the assessment of land capacity to support primary production^9^, is a key tool for land use planning^10^. It can provide useful information to policymakers, stakeholders, and land managers and guide their decisions and planning strategies.

Over the last couple of years, LSA has been extensively used in New Zealand (NZ) to assess the suitability of several agricultural products. For example, Harris et al.^12^ performed an LSA combining biophysical feasibility, yields, economic returns and economic importance information to evaluate the productive potential of the main agriculture sectors (i.e., horticulture and viticulture, arable, dairy, forestry, and sheep & beef). LSA was also used to assess climate change effects on perennial crop growing conditions^13,14^, showing that for horticulture – kiwifruit, apple, cherry, avocado, blueberry and viticulture (Sauvignon blanc and Pinot noir) – both threats (e.g., satisfying winter chill requirement) and opportunities (e.g., warmth during the growing season) are expected in the coming decades^13^. As part of a study on the potential role of high-value crops (i.e., manuka honey, chestnuts, truffles, peas, potatoes and onions) in reducing biogenic greenhouse gas emissions, Thomas et al.^15,16^ performed LSA modelling, revealing that the availability of suitable land is unlikely to force the transition to new crops. Following the methodology of Vetharaniam et al.^13,14^, LSA was applied to almonds, a currently marginal agricultural product in NZ, highlighting potential good growing conditions under climate change^17^.

Although the land suitability of the most important agricultural products in NZ has been assessed, some gaps and challenges remain. Among them, the most important is the differences in methodologies employed for LSA for various crops in NZ. Qualitative^15,16^ and fuzzy-logic approaches^13,14,17^ were used to convert environmental parameters into suitability values in LSA. Moreover, overall suitability does not always refer to the same concept (i.e., limiting factor vs. geometric mean). These methodology differences are a major weakness of the land suitability information currently available for NZ.

There is also a necessity to perform LSA under changing climate conditions^10^. In NZ, only some of the previous LSAs have explored the evolution of suitability under climate change^13,17^; thus, suitability projections are not available for several agricultural products^15^. On the other hand, LSAs under climate change projection^13,17^ have not assessed climate-related uncertainty of suitability, which is important to consider given the major role of climate variability on agricultural production^1^. Moreover, these studies were conducted using CMIP5 climate data projections, whereas CMIP6 data are now available and have shown high differences in crop productivity and more pronounced impacts of climate change with CMIP6 than the previous generation^3^. Updating LSA with the new generation of climate data is necessary as climate modelling has improved, leading to changes in projected impacts on agriculture.

Finally, the last critical gap is the fact that previous LSA have not considered crop water requirements (CWR). Irrigation is extensively used in NZ to satisfy CWR^18^. However, previous LSAs assumed water requirements are met with irrigation but without providing information about the additional water needs^15^ or did not consider the possibility of irrigation use, assuming precipitation as the only water supplier for crops^13,14^. While climate change affects water availability^19^, it is also essential to consider irrigation options for future water management planning.

This study aims to fill the methodological gaps in current LSA’s by developing a new, widely applicable framework and understanding how growing suitability patterns are projected to evolve in NZ under different climate change scenarios. This work focuses on four crops – apple (*Malus* spp.), cherry (*Prunus avium*), maize (*Zea mays*) and wheat (*Triticum* spp.)– that are widely grown in NZ and represent a diversity of growing requirements. Although socio-economic aspects have been integrated into previous LSA’s^10,12,20,21^, this work only focuses on agroclimatic suitability, referring to environmental constraints (i.e., soil and climate).

## Method and data

### Study site

About half of NZ’s land area is dedicated to agriculture and forestry (see Supplementary Fig. S1) – 40% for livestock pasture, 8% planted in forests and 2% for cropping and horticulture^22^. It represented a significant part (81.8%) of NZ’s exports in the year to March 2023, corresponding to an export revenue of 57.2 billion NZ$ and making agriculture a pillar of NZ’s economy. NZ agricultural production is divided into seven sectors: dairy (44%), meat and wool (22%), forestry (12%), horticulture (12%), arable (0.5%) and processed food and other products (6%)^11^. The impacts of climate change on pasture, water resources, and animal heat stress highly threaten dairy and meat production, the most economically profitable agricultural sectors. However, the highly profitable, very localised horticulture production and its long-term growing processes make this sector especially vulnerable to rapidly changing climate conditions.

### Data

Data used for the land suitability analysis included climate, soils, land use capability, and slope are presented in Table 1. The climate data were extracted from the NEX-GDDP-CMIP6 (NASA Earth Exchange Global Daily Downscaled Projection) dataset^23^, which provides global daily and bias-corrected data derived from CMIP6^24,25^. The dataset covers the 1950–2100 period with high spatial resolution (0.25°) for 35 General Circulation Models (GCMs) and four Share-Socioeconomic Pathways (SSP1-2.6; SSP2-4.5, SSP3-7.5 and SSP5-8.5) corresponding to low, moderate, intermediate and high emissions scenarios^26^. Data were obtained for the four SSPs and five GCMs (NorESM2-MM, GFDL-ESM4, EC-Earth3, ACCESS-CM2, CNRM-CM6-1) following the six models selected by Gibson et al.^27^ to downscale CMIP6 data over NZ. Note that the AWI-CM-1-1MR is not available in the NEX-GDDP-CMIP6 dataset.

The New Zealand Fundamental Soil Layer (FSL) database provided the potential rooting depth (PRD), profile available water (PAW), salinity, drainage class and stone content information. The land use capability (LUC) and slope data came from the New Zealand Land Resource Inventory (NZLRI) and the Land Environment of New Zealand (LENZ) database. Soil data were resampled to a 0.05° spatial resolution to match NEX-GDDP-CMIP6 data.

**Table 1:** Details of the data used for the land suitability analysis.

Name	Source	Format	Spatial Resolution
**Climate**			
NEX-GDDP-CMIP6	Thrasher et al.^23^	NetCDF	0.25° x 0.25°
**Soil**			
New Zealand Fundamental Soil Layer (NZFSL)	10.7931/L1B36 10.26060/GDBJ-S770 (access date January 2024)	ESRI Shapefile	1:50,000
Land Use Capability	10.26060/P7AG-AN36 (access date January 2024)	ESRI Shapefile	1:50,000
Slope	McCarthy et al.^28^	GeoTIFF	100 m x 100 m

### Land suitability analysis (LSA)

#### LSA Modelling – LSAPy

A wide range of LSA approaches exists, from traditional qualitative methods to more sophisticated ones like machine learning^10,21,29^. To ensure consistency, the choice of approach was reduced to those already used in previous LSA studies in NZ. The qualitative approach used by Thomas et al.^15^ was excluded because discrete values don’t capture the continuity of environmental conditions that may lead to limitations and inaccuracies^10,13,21^. Thus, the fuzzy-logic approach proposed by Vetharaniam et al.^13,14^ was used.

The fuzzy logic approach was programmed in Python, named LSAPy (Land Suitability Analysis in Python), and used to conduct the LSA. LSAPy encompasses several modules operating together to perform spatial and temporal LSA^30^. The package provides an easy workflow to manage the large number of suitability criteria and supports several methods to combine them.

**Fig. 1:**
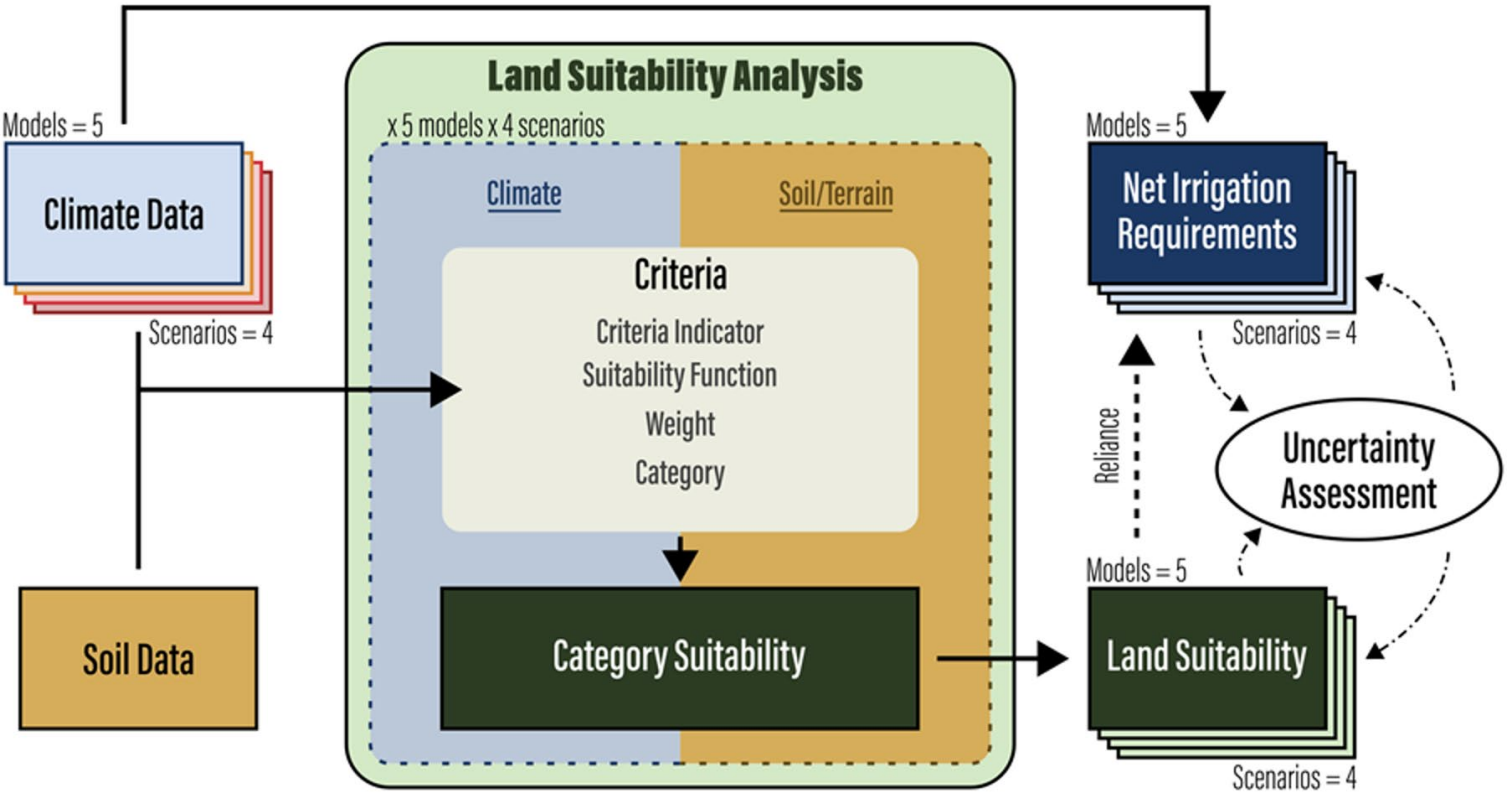
Workflow of the Land Suitability Analysis, Net Irrigation Requirements, and Uncertainty modelling approaches. Climate data were used to assess the Net Irrigation Requirements (NIR) and were combined with soil data to perform the Land Suitability Analysis for each model and scenario. The LSA was conducted based on a set of criteria (climate or soil/terrain) relevant to the studied crop. The land suitability was obtained by first aggregating the criteria suitability into category suitability and then aggregating the latter to obtain the overall land suitability. The outputs of individual models were used to assess the uncertainty of the NIR and land suitability.

The LSAPy workflow is presented in the Fig. 1, and starts with the definition of the crop requirement criteria. Each criterion is defined with an associated indicator that is used to compute its suitability; for example, a *Growth Temperature Requirement* criterion will be associated with a *Growing Degree Day (GDD)* indicator. Besides the indicator, a function is defined to compute the criterion’ suitability by converting the indicator to suitability values. For discrete or categorical indicators, the function corresponds to a simple rule attributing a suitability value to each indicator value or category. For continuous indicators, membership functions (sigmoid-like or Gaussian-like) are applied to obtain suitability values ranging from 0 (totally unsuitable) to 1 (very highly suitable). Other optional attributes, such as criteria weight and category, can also be defined to conduct more specific and complex LSA.

LSAPy then computes the individual criteria suitability and combines them to provide the overall land suitability (Fig. 1). The final suitability can be calculated as the mean, weighted mean, geometric mean, weighted geometric mean or limiting factor of all the criteria.

The following summary terms were used to describe suitability values: *unviable* for 0-0.6, *viable* for 0.6–0.9 and *excellent* for 0.9-1; where adaptions would likely be required to successfully grow crops in *viable* locations, while *excellent* ones would require only minor or no adaptations^14,31^.

#### LSA Criteria

A crop-specific criteria approach was used to better consider the different requirements of each crop. The criteria were divided into two categories, the soil-related and climate-related criteria. The criteria used for apples and cherries LSA came from Vetharaniam et al.^13,14^ and include potential rooting depth (PRD), drainage, slope and land use capability (LUC) for the soil, winter chill, frost risk, temperature for maturation, fruit size (apples) and sunburn (apples) for the climate. The PRD, profile available water (PAW), drainage, slope, salinity and stone content (maize) were used as soil criteria for maize and wheat LSA; the rainfall excess, frost risk, temperature for maturation and heat risk (wheat) for climate following Thomas et al.^15,16^ application. The indicators related to climate criteria were computed using the *xclim* Python package^32^, and more details about the criteria, their associated indicator and membership functions (equations and parameters) are provided in Supplementary Methods. The membership functions used to compute wheat suitability criteria are presented as examples in Supplementary Figure S2.

The soil-related criteria were combined using a weighted geometric mean to provide the soil suitability (Eq. 1). The climate suitability was computed in the same way.

1 $$\:{S}_{cat}=\:{\left(\prod\:_{c=1}^{n}{{S}_{c}}^{{w}_{c}}\right)}^{\frac{1}{{w}_{cat}}},\:\:with\:{w}_{cat}=\sum\:_{c=1}^{n}{w}_{c}$$

where S_cat_ is the suitability of the category, S_c_ the suitability of the criteria, w_c_ its associated weight and n the number of criteria.

Finally, the overall land suitability was computed using the weighted geometric mean of the soil and climate suitability, with the sum of climate-related criteria weights as weight for climate and the sum of soil-related criteria weights as weight for soil (Eq. 2).

2 $$\:S=\:{\left({{S}_{st}}^{{w}_{st}}\:\times\:\:{{S}_{cli}}^{{w}_{cli}}\right)}^{1/({w}_{st}+\:{w}_{cli})}$$

where S is the land suitability, S_st_ and S_cli_ the suitability of soil and terrain, and climate categories respectively, and w_st_ and w_cli_ their associated weights.

The future land suitability of crops assumes that changes in future suitability will be mainly driven by changes in climate conditions. Thus, the same indicators for soil and terrain criteria were used for the historical and future periods.

### Water requirements

#### Crop water requirements

The crop water requirements were calculated over the growing season with a daily time step following the method described by Allen et al.,^33^ and using Eq. (3).

3 $$\:CWR={ET}_{0}\:\times\:\:{K}_{c}$$

where CWR is the crop water requirement (mm), K_c_ is the crop coefficient, and ET_0_ (mm) is the reference evapotranspiration^34^ as:

4 $$\:{ET}_{0}=0.0023\:\times\:Ra\:\times\:\left(T+17.8\right)\times\:\:\sqrt{Tmax-Tmin}$$

where Ra is the extraterrestrial solar radiation (kg/MJ), T, Tmin and Tmax are respectively the mean, minimum and maximum temperature (°C).

The crop coefficient (K_c_) curve was built for each growing season based on the length of the crops’ development stages and their associated crop coefficient (K_c_) described in Supplementary Table S6, according to Allen et al.^33^ – an example of a K_c_ curve for wheat is presented in the Supplementary Figure S3. The K_c_ value can be adjusted to obtain more accurate estimates^33^. The adjustment of the initial stage crop coefficient is made based on wetting events, considering both precipitation and irrigation. However, given the difficulty of aggregating irrigation practices at the spatial resolution of climate data, no adjustments were made for K_c init_. The K_c_ for the mid-season were adjusted (Eq. 5) considering the climate conditions (i.e., humidity and wind), but not for the frequency of the wetting event for the same reason as the initial stage.

5 $$\:{K}_{c\:adj\:}=\:{K}_{c}+\:\left[0.04\:\times\:\left({u}_{2}-2\right)-\:0.004\:\times\:\left({RH}_{min}-\:45\right)\right]\:\times\:\:{\left(\frac{h}{3}\right)}^{0.3}$$

where K_c adj_ is the adjusted K_c_ value, K_c_ is the base value, u_2_ is the mean value for daily wind speed (m/s) at 2 m height over the mid-season stage, RH_min_ mean value for daily minimum relative humidity (%) during the mid-season stage and h the plant height (m) during mid-season stage, with:

6 $$\:{RH}_{min}=\:\frac{e^\circ\:\left(Tmax\right)}{e^\circ\:\left(Tmin\right)}\times\:100$$

where e°(Tmax) and e°(Tmin) are respectively the saturation vapour pressure at maximum and minimum daily air temperature.

Finally, the K_c_ of the end season were also adjusted to climate conditions using Eq. 5 for values less than 0.45^33^.

The start of the growing season corresponds to the sowing date or to the ”green-up date” for perennial crops (i.e., the time when the initiation of new leaves occurs)^33^. As a simple approximation, the start of the growing season was set to a fixed date for each crop. Two different dates were used for wheat to represent the winter and spring varieties, and set as the 15th of May for winter wheat and the 15th of August for spring wheat. For maize, the start of the growing cycle was set to the 1st of September, while it was set to the 15th of October for apple and cherry. Those dates were chosen based on the average date of the day of full bloom (apple) and budbreak (cherry) modelling over the 21st century, and to be representative of current practices in New Zealand across the different varieties for maize and wheat.

#### Net Irrigation Requirements

The NIR was computed for the crop growing season following the method used by Oumarou Abdoulaye et al.^35^ and the CROPWAT approach^36^ as:

6 $$\:\begin{array}{c}{IR}_{net}=CWR-{P}_{eff}\:if\:CWR>\:{P}_{eff}\\\:{else\:IR}_{net}=0\end{array}$$

Where CWR is the crop water requirements and P_eff_ is the effective precipitation (mm). Effective precipitation depends on several parameters and processes such as run-off, percolation, soil moisture, and rooting depth, making estimating its value difficult, especially with low spatial resolution data. Thus, the simple USDA soil conservation method was used and calculated for the daily value as:

7 $$\:\begin{array}{c}{P}_{eff}=\:\frac{P\:\times\:\:\left(4.17-0.2\:\times\:P\right)}{4.14}\:for\:P<8.2\\\:{P}_{eff}=4.17+0.1\:\times\:P\:for\:P\:\ge\:8.3\end{array}$$

where P is the daily precipitation.

The CWR requirement and NIR were then aggregated annually.

### Uncertainty in future changes

The uncertainty in future projected changes in suitability and NIR has been assessed, based on the outputs of individual climate models (Fig. 1), using *xclim*^32^ and following the approach defined by IPCC^37^. Three categories are used to describe the future changes: *robust signal* where more than 66% of models show change greater than variability and more than 80% of all models agree on the sign of change; *no change or no robust signal* where less than 66% of model show change greater than variability, and *conflicting signals* where more than 66% of models show change greater than variability but less than 80% of all models agree on the sign of change.

The changes were computed for the near term (2010–2039), mid-term (2040–2069), and long term (2070–2099) using the 1980–2009 baseline. Note that the baseline differs from the one used by the IPCC^37^(i.e., 1995–2014) in order to integrate a 30-years spanning period.

## Results

### Agroclimatic suitability

#### Historical and future suitability

**Fig. 2:**
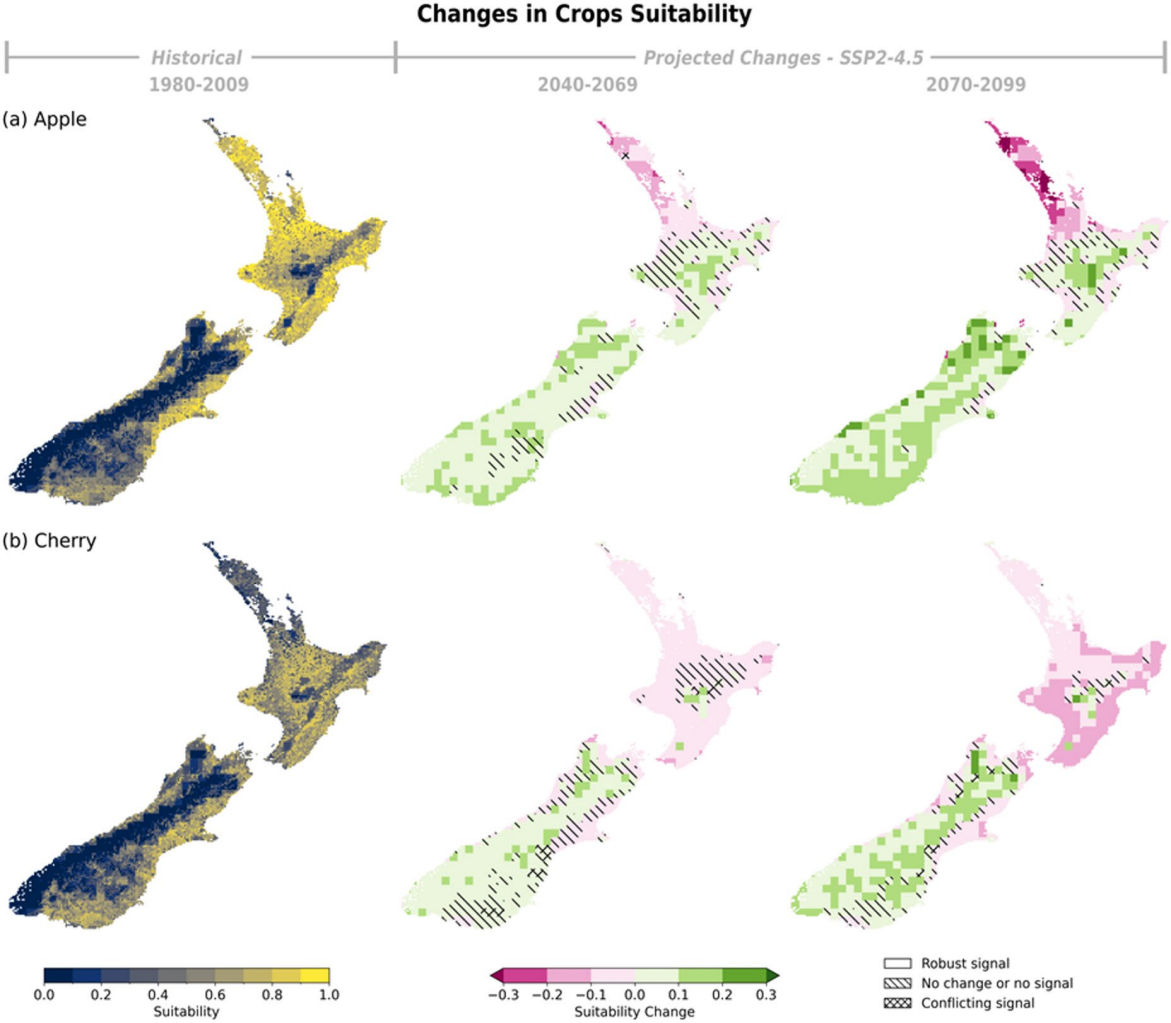
Historical crops suitability and mid- and long-term changes. Displayed are projected spatial patterns of multi-model (*n* = 5) mean change in crop suitability with SSP2-4.5 in 2040–2069 and 2070–2099 relative to the 1980–2009 historical suitability for **(a)** apple and **(b)** cherry. The overlay represents the robustness^35^: no overlay indicates areas where the change is robust; diagonal lines indicate areas with no change or no robust significant change; crossed lines indicate areas of conflicting signals — maps generated in Python using matplotlib 3.9.2 (https://matplotlib.org).

**Fig. 3:**
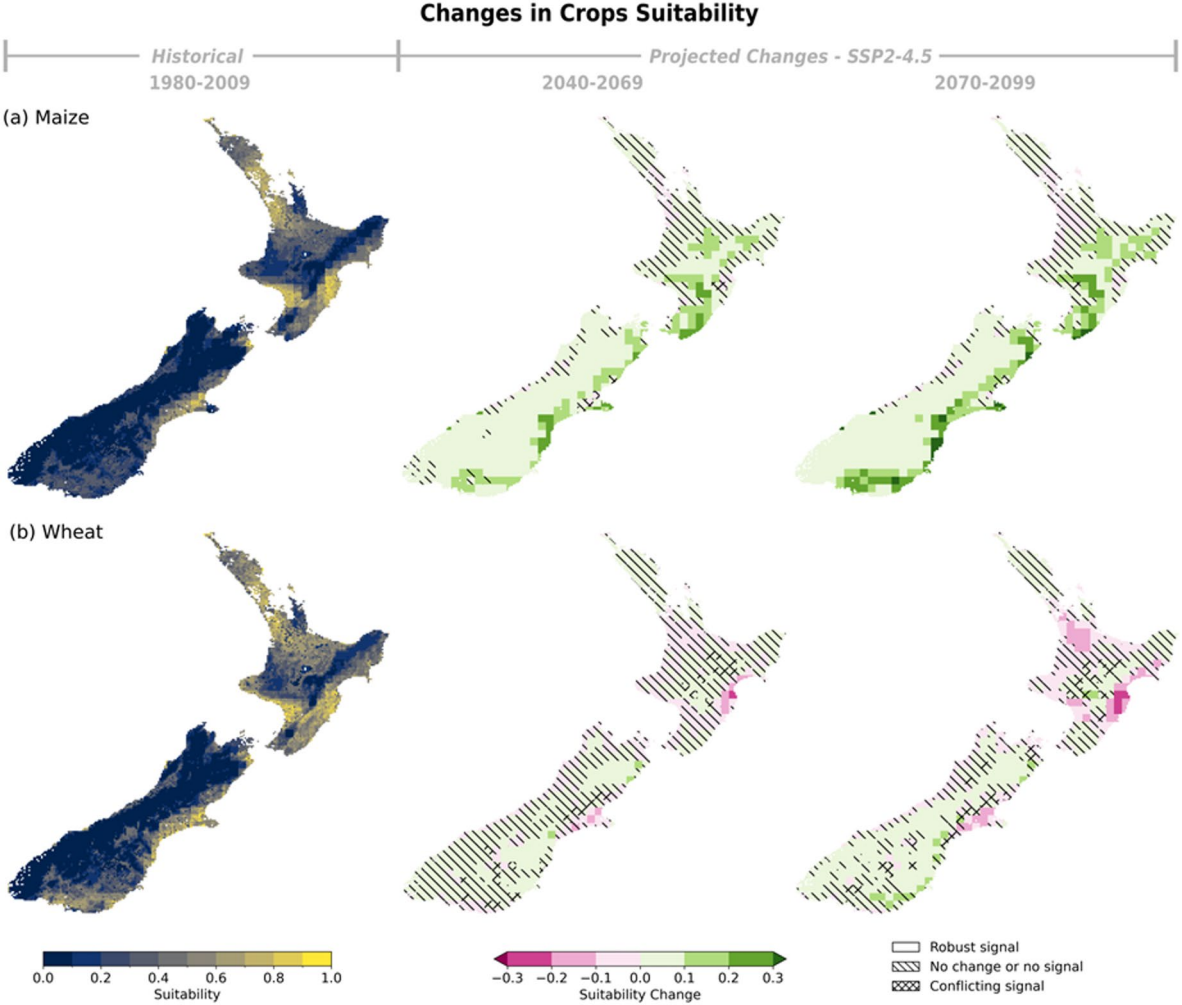
Historical crops suitability and mid- and long-term changes. Displayed are projected spatial patterns of multi-model (*n* = 5) mean change in crop suitability with SSP2-4.5 in 2040–2069 and 2070–2099 relative to the 1980–2009 historical suitability for **(a)** maize and **(b)** wheat. The overlay represents the robustness^35^: no overlay indicates areas where the change is robust; diagonal lines indicate areas with no change or no robust significant change; crossed lines indicate areas of conflicting signals — maps generated in Python using matplotlib 3.9.2 (https://matplotlib.org).

The historical suitability and projected changes in the crops’ suitability are shown for the mid- and long-term in Figs. 2 and 3 for SSP2-4.5 (see Supplementary Figures S5 for near-term SSP2-4.5, and S6, S7 and S8 for SSP1-2.6, SSP3-7.0 and SSP5-8.5). The regional and national suitability areas are provided in Table 2 and Supplementary Table S7 for SSP2-4.5 and SSP5-8.5 (see Supplementary Tabs. S8-S9 for SSP1-2.6 and SSP3-7.0).

Similar spatial patterns are observed for the historical suitability of apple and cherry, but with varying intensity (Fig. 2). The national historical *viable* suitability areas are relatively close for both crops, with 79,650 km^2^ for apple and 75,325 km^2^ for cherry, but apple also has 18,600 km^2^ of *excellent* suitability area, whereas cherry has none (Table 2).

Maize and wheat also have relatively similar historical suitability spatial patterns (Fig. 3). Generally, wheat presents a larger *viable* and *excellent* suitability area than maize at the regional level (except Hawke’s Bay), reflected at the national scale with an area of 37,425 km^2^ and 700 km^2^ for wheat, compared to 22,150 km^2^ and 200 km^2^ for maize (Table 2).

Regarding the projected suitability, although the intensity of projected changes varies across space and time depending on the SSP, the results show a general trend in the changes for each crop being more pronounced toward the end of the century.

Land suitability for apples decreases in the future in the upper part and the periphery of the North Island and increases in the rest of the country, with changes mostly ranging between − 0.2 and 0.2 (Fig. 2 and Supplementary Figs. S5-S8). Hawke’s Bay, the region having the most important apple production area^38^, is expected to benefit from an increase in *viable* suitability area under all SSPs for both mid- and long-term (Table 2 and Supplementary Tabs. S7-S9). However, its *excellent* suitability area is expected to decrease in the mid-term for SSP3-7.0 and SSP5-8.5, and in the long-term for all SSPs except SSP1-2.6. Tasman, the second region with the largest apple production area, would benefit from an increase in *viable* and *excellent* suitability under all SSPs in the mid- and long-term (Table 2 and Supplementary Tabs. S7-S9). In NZ, the *viable* and *excellent* suitability are projected to increase for all SSPs in the mid-term, to 86,270 km^2^ and 21,325 km^2^ under SSP2-4.5, and 89,835 km^2^ and 19,025 km^2^ under SSP5-8.5.

The suitability of cherry is projected to decrease across the North Island, the north of the South Island, and the Canterbury region, while increasing in the rest of the South Island (Fig. 2 and Supplementary Figs. S5-S8). While a decrease in *viable* suitability area is projected for almost all the regions of NZ, an opposite trend is projected for Otago, the main producer of cherries^38^, and likewise for the Southland region (Table 2 and Supplementary Tabs. S7-S9). No changes are projected regarding the *excellent* suitability area, remaining zero for all regions and scenarios in the mid- and long-term. At the national level, the *viable* suitability area is projected to decrease from 75,325 km^2^ to 70,100 km^2^ in the mid-term with SSP2-4.5, and up to 48,400 km^2^ in the long-term with SSP3-7.0 (Table 2 and Supplementary Tab. S8).

The results show that the suitability of maize increases in the future across the country and for all SSPs (Fig. 3 and Supplementary Figs. S5-S8). The main regions currently growing maize in NZ are Manawatū-Whanganui, Waikato, Gisborne, Hawke’s Bay, Bay of Plenty, and Northland^38^; the *viable* suitability area in these regions is expected to increase, with the most important one occurring for Manawatū-Whanganui (Table 2 and Supplementary Tabs. S7-S9). However, the *excellent* suitability of those regions remains small or null for mid and long-term for all SSPs. Among the other regions, Canterbury, Otago and Southland are those that could benefit the most in the future with a net increase of their *viable* suitability areas, and *excellent* suitability areas for the first two. For NZ, the *viable* and *excellent* suitability areas are projected to increase up to 61,590 km^2^ and 2210 km^2^, respectively, in the long-term under SSP5-8.5 (Table 2).

Finally, a small increase in the wheat suitability is projected in the future in the South Island, while the projected changes are more contrasted for the North Island (Fig. 3 and Supplementary Figs. S5-S8). Canterbury, the main region producing wheat^38^, is expected to see its *viable* suitability area increasing in mid and long-term for all SSPs, except SSP2-4.5 in mid-term and SSP5-8.5 in long-term (Table 2 and Supplementary Tabs. S7-S9). An increase in *excellent* suitability area in Canterbury is also projected in the mid-term for all SSPs, but only under SSP1-2.6 in the long-term. Apart from Canterbury, Otago is the region that is projected to benefit the most from increasing suitability areas. The *viable* suitability area in NZ is projected to decrease in mid- and long-term for SSP2-4.5 and SSP5-8.5 (Table 2) but is expected to increase under SSP1-2.6 and SSP3-7.0 (Supplementary Tabs. S7-S8). The *excellent* suitability area is projected to increase except in the long-term for SSP3-7.0 and SSP5-8.5.

### Uncertainty in projected suitability

The projected changes show the multi-model agreement varies depending on crop, period and SSP. In general, the model agreement is better in the long-term than in near- and mid-term, with a greater extent of *robust signal*, especially for SSP2-4.5 and SSP5-8.5 (Figs. 2 and 3 and Supplementary Figs. S5 and S8), while the changes in robustness are less important for SSP1-2.6 and SSP3-7.0 (Supplementary Figs. S6 and S7). However, it’s also in the long-term that the number of *conflicting signals* is the most important, although remaining limited.

In the near-term, the crops present a greater extent of *robust signal* only under SSP1-2.6 and SSP3-7.0 (Supplementary Figs. S6 and S7), while a majority of *no change or no robust signal* is identified for SSP2-4.5 and SSP5-8.5 (Supplementary Figs. S5 and S8). However, from mid-term, a majority of *robust signals* are projected for all the crops except wheat, with small patches of *no change or no robust signals* (Figs. 2 and 3 and Supplementary Figs. S6-S8). In the mid- and long-term, the signal is *robust* for apple in the north and south of the North Island, and the majority of the South Island. Cherry has similar patterns with a greater and smaller extent of *robust signal* on the North Island and South Island, respectively. A *robust signal* is observed for almost all of the South Island for maize, while remaining located in the centre and south of the North Island. Finally, the location of the *robust signal* is more disparate for wheat, although it is extensively present in the long-term.

The suitability area multi-model standard deviations provided in Table 2 and Supplementary Table S7-S9 are relatively large compared to the multi-model mean, the latter remaining in the standard deviation range of the historical value for most of the cases. In addition, although results don’t show a clear difference in uncertainty between crops at the regional level, a national trend emerges with, proportionally to the multi-model mean, higher uncertainty for wheat and maize than for apple and cherry. For the *viable* suitability area, the uncertainty represents around 35% of the multi-model mean compared to around 5% and 5–10% for apple and cherry (Table 2 and Supplementary Tab. S9). Moreover, the uncertainty tends to be higher for the *excellent* than *viable* suitability area, with values corresponding to around 40% of the multi-model mean for apple, more than 150% for cherry, and around 60–80% for maize and wheat. The higher uncertainty value for the later crops is thus confirmed, noting that cherry is the highest only because of its really small area under *excellent* suitability (Table 2 and Supplementary Tab. S9).

**Table 2:** Historical and future mid-term crops suitability areas (km^2^). Presented are the historical (1980–2009) and future mid-term (2040–2069) multi-model (*n* = 5) mean suitability areas in km^2^ for SSP2-4.5 and SSP5-8.5 for apple, cherry, maize and wheat – in brackets are the corresponding multi-model standard deviations. The suitability areas are presented for each of the 16 New Zealand administrative regions and total of New Zealand. The suitability area is divided into two categories: viable (V) areas, where suitability ranges between 0.6 and 0.9, and excellent (E) areas, where suitability ranges between 0.9 and 1. For each region, the potential agricultural area (PAA) in km^2^ is given, and statistics were computed only on this one, excluding areas unavailable for agriculture (see Spatial Statistical Analysis in Supplementary Methods). The font represents the sign of change compared to the historical areas, with Italic highlighting an increase, Bold a decrease and bolditalic no change.

Region			Apple			Cherry			Maize			Wheat		
ID	PAA		Historical	Mid term		Historical	Mid term		Historical	Mid term		Historical	Mid term	
				SSP2-4.5	SSP5-8.5		SSP2-4.5	SSP5-8.5		SSP2-4.5	SSP5-8.5		SSP2-4.5	SSP5-8.5
NTL	7425	V	5200	**4575 (914)**	**3480 (1304)**	***0***	***0***	***0***	1850	*2320 (419)*	*2565 (680)*	2900	*3415 (255)*	*3600 (422)*
		E	1500	**375 (546)**	**115 (243)**	***0***	***0***	***0***	***0***	***0***	***0***	50	50 (0)	50 (0)
AUK	2825	V	1950	**1615 (609)**	**1175 (626)**	250	**50 (161)**	**15 (22)**	2350	*2450 (143)*	*2420 (164)*	2425	*2490 (76)*	**2405 (149)**
		E	700	**205 (195)**	**60 (72)**	***0***	***0***	***0***	***0***	***0***	***0***	***0***	***0***	***0***
WKO	17,125	V	10,300	*11,715 (1458)*	*12,570 (868)*	10,675	**8235 (528)**	**7335 (268)**	3125	*4575 (1603)*	*4200 (1637)*	5700	**5340 (1965)**	**4195 (1126)**
		E	5250	**3345 (1287)**	**2120 (719)**	***0***	*5 (11)*	***0***	***0***	***0***	***0***	***0***	***0***	***0***
BOP	5850	V	3150	*3675 (360)*	*4260 (174)*	4275	**3720 (288)**	**3345 (140)**	***0***	*230 (219)*	*300 (346)*	1100	*1290 (570)*	**850 (278)**
		E	2200	**1875 (508)**	**1295 (266)**	***0***	***0***	***0***	***0***	***0***	***0***	***0***	***0***	***0***
GIS	6100	V	4125	*4145 (247)*	*4355 (290)*	2800	**1705 (391)**	**1325 (409)**	225	310 (224)	370 (223)	375	**365 (68)**	**335 (104)**
		E	1600	*1780 (247)*	**1570 (290)**	***0***	***0***	***0***	***0***	***0***	***0***	***0***	***0***	***0***
HKB	10,000	V	6300	*6410 (700)*	*7055 (502)*	6550	**5135 (464)**	**4675 (558)**	4900	*5225 (1078)*	*5270 (1103)*	4750	**2770 (920)**	**2390 (689)**
		E	2650	*2905 (1030)*	**2360 (693)**	***0***	***0***	***0***	125	**100 (43)**	125 (0)	50	**10 (14)**	**0**
TKI	4525	V	2625	**2085 (555)**	*3005 (618)*	3525	**3225 (268)**	**2745 (485)**	250	*350 (110)*	*335 (111)*	1700	*1795 (504)*	**1525 (462)**
		E	1775	*2365 (555)*	**535 (485)**	***0***	***0***	***0***	***0***	***0***	***0***	25	**15 (14)**	**0**
MWT	16,000	V	12,825	**11,745 (1221)**	**11,920 (619)**	9575	**8225 (346)**	**7325 (327)**	4750	*7440 (2348)*	*7725 (2668)*	5700	**5380 (2123)**	**4805 (2089)**
		E	1025	*2545 (1735)*	*2520 (1274)*	***0***	***0***	***0***	***0***	*45 (41)*	*30 (27)*	225	**150 (112)**	**60 (72)**
WGN	5175	V	4550	**3890 (484)**	**3770 (326)**	3500	**2375 (467)**	**550 (589)**	525	*2780 (1067)*	*3240 (816)*	1600	*2245 (1006)*	*2105 (392)*
		E	450	*1165 (498)*	*1295 (332)*	***0***	***0***	***0***	***0***	***0***	***0***	***0***	***0***	***0***
TAS	2700	V	1725	*1920 (86)*	*1955 (99)*	1575	**1520 (147)**	**1455 (101)**	0	*10 (14)*	*5 (11)*	25	*70 (69)*	**20 (11)**
		E	100	*300 (151)*	*380 (122)*	***0***	***0***	***0***	***0***	***0***	***0***	***0***	***0***	***0***
MBH	4825	V	2600	*2695 (124)*	*2770 (108)*	2100	*2110 (141)*	**2045 (163)**	625	*1155 (246)*	*1200 (222)*	875	*930 (269)*	**790 (244)**
		E	125	*470 (278)*	*520 (195)*	***0***	***0***	***0***	75	*120 (60)*	*140 (49)*	50	50 (0)	**40 (22)**
NSN	200	V	125	*150 (25)*	*165 (14)*	100	**80 (11)**	**80 (11)**	***0***	***0***	***0***	***0***	***0***	***0***
		E	25	25 (0)	25 (0)	***0***	***0***	***0***	***0***	***0***	***0***	***0***	***0***	***0***
WTC	2200	V	1200	*1275 (18)*	*1300 (25)*	1150	*1155 (124)*	**1045 (203)**	25	25 (0)	25 (0)	25	25 (0)	25 (0)
		E	0	*230 (227)*	*280 (182)*	***0***	***0***	***0***	***0***	***0***	***0***	***0***	***0***	***0***
CAN	32,300	V	14,825	*15,930 (1690)*	*16,975 (1781)*	15,325	*16,205 (3728)*	*16,585 (3742)*	3525	*7980 (4831)*	*9185 (4421)*	5925	**5865 (4586)**	*6210 (4415)*
		E	1200	*2850 (2312)*	*3120 (2214)*	***0***	*65 (132)*	*15 (34)*	***0***	*415 (596)*	*700 (603)*	250	*735 (897)*	*500 (604)*
OTA	26,300	V	3825	*8035 (4001)*	*8900 (4447)*	7225	*9060 (2962)*	*9375 (3297)*	***0***	*990 (1193)*	*1670 (1606)*	1700	*2415 (2266)*	*2650 (2402)*
		E	***0***	*285 (390)*	*415 (533)*	***0***	***0***	***0***	***0***	*5 (11)*	*25 (43)*	50	*315 (340)*	*235 (254)*
STL	13,750	V	4325	*6410 (2008)*	*6865 (1954)*	6700	*7300 (1449)*	*7280 (1516)*	***0***	*820 (1632)*	*1430 (2260)*	2625	**2480 (2781)**	2625 (2691)
		E	***0***	*605 (1072)*	*820 (1336)*	***0***	***0***	***0***	***0***	***0***	***0***	***0***	*290 (399)*	*180 (337)*
NZ	157,375	V	79,650	*86,270 (3263)*	*89,835 (4108)*	75,325	**70,100 (6554)**	**66,480 (7463)**	22,150	*36,660 (13096)*	*39,940 (14329)*	37,425	**36,885 (13202)**	**34,530 (12889)**
		E	18,600	*21,325 (8755)*	*19,025 (7308)*	***0***	*70 (130)*	*15 (34)*	200	*685 (720)*	*880 (712)*	700	*1230 (988)*	*1065 (874)*

Regions: Northland (NTL), Auckland (AUK), Waikato (WKO), Bay of Plenty (BOP), Gisborne (GIS), Hawke’s Bay (HKB), Taranaki (TKI), Manawatū-Whanganui (MWT), Wellington (WGN), West Coast (WTC), Canterbury (CAN), Otago (OTA), Southland (STL), Tasman (TAS), Nelson (NSN), Marlborough (MBH), New Zealand (NZ).

### Net irrigation requirements

**Fig. 4:**
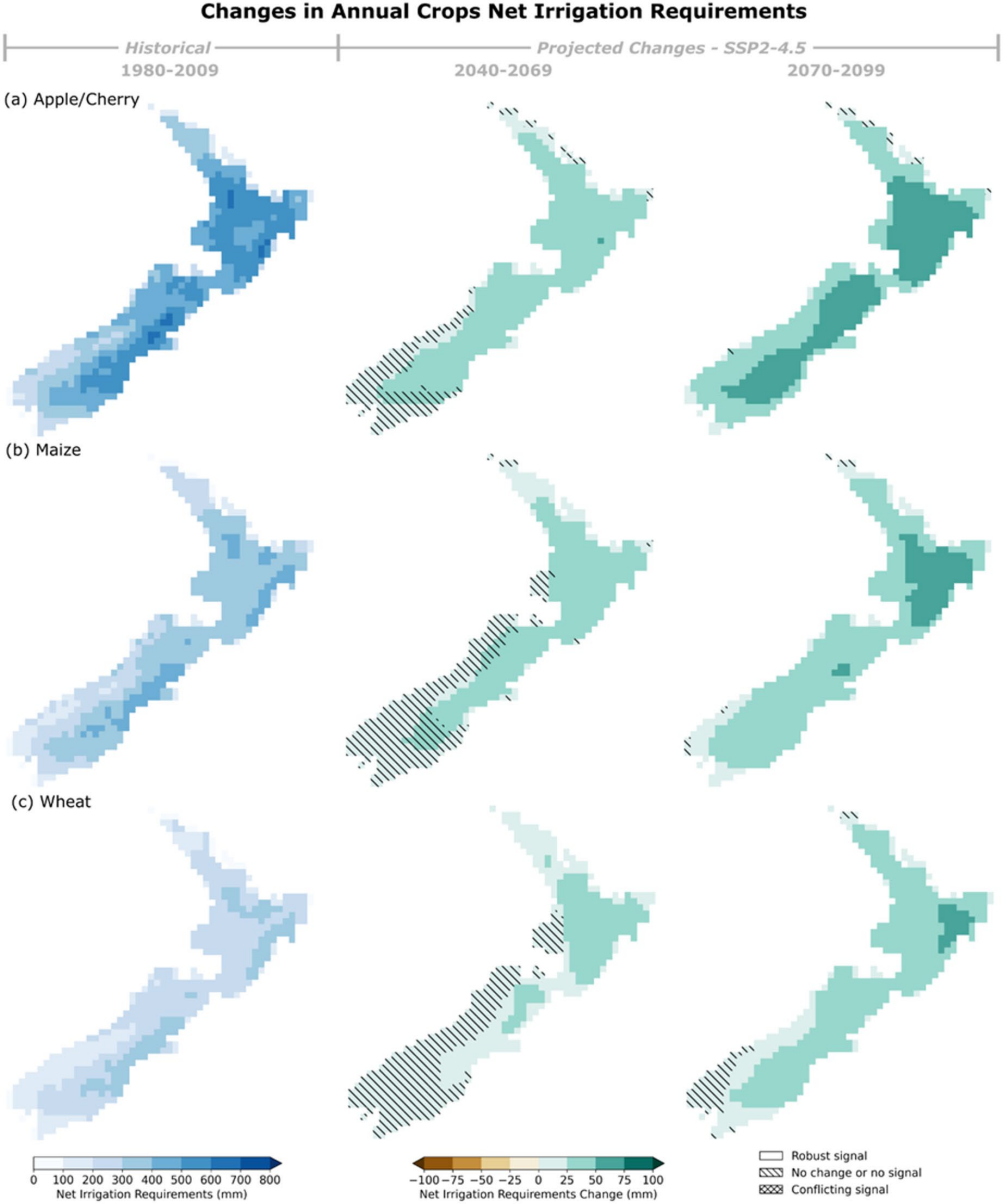
Historic annual crops net irrigation requirements (NIR) and mid- and long-term changes. Displayed are projected spatial patterns of multi-model (*n* = 5) mean change in annual crop net irrigation requirements (NIR) with SSP2-4.5 in 2010–2039, 2040–2069 and 2070–2099 relative to the 1980–2009 historic NIR **(a)** apple and cherry, **(b)** maize, **(c)** wheat. The overlay represents the robustness (IPCC, 2021): no overlay indicates areas where the change is robust; diagonal lines indicate areas with no change or no robust significant change; crossed lines indicate areas of conflicting signals — maps generated in Python using matplotlib 3.9.2 (https://matplotlib.org).

The mid- and long-term spatial projected changes in annual net irrigation requirements (NIR) are presented in Fig. 4 for SSP2-4.5 (see Supplementary Figs. S5 for near-term SSP2-4.5, and S9, S10 and S11 for SSP1-2.6, SSP3-7.0 and SSP5-8.5). The regional statistics of annual NIR are presented in Supplementary Figures S12, S13 and S14 for the near-, mid- and long-term.

The historical NIR are higher for apple and cherry, with values ranging mostly between 200 and 600 mm/year, compared to 200–500 mm/year for maize and 100–400 mm/year for wheat (Fig. 4 and Supplementary Figs.S5 and S9-S14). For each crop, the NIR tend to be higher for the North Island and the Canterbury region, while the south-west of the South Island presents the lowest NIR.

The NIR increase in all the regions under the SSP2-4.5 and SSP5-8.5 (Supplementary Figs. S12-S14), but with *no changes or robust signals* projected in the near-term (Supplementary Figs. S5 and S11). In the mid-term, an increase of 25–50 mm/year is projected for all the crops and most of the country under SSP2-4.5, except the West Coast and the lower part of the South Island where *no change or no robust signals* are projected (Fig. 4); for the SSP5-8.5, the area with *no change or no robust signals* is smaller and the NIR range between 25 and 75 mm/year (Supplementary Fig. S11). In the long-term, *robust* changes are projected for the entire country for both SSPs and all the crops. The NIR increase is higher for apple and cherry – 25–75 mm/year for SSP2-4.5 and 50 to > 100 mm/year for SSP5-8.5 – with the most important increase occurring in the central and south of the North Island, and in the north and south of Canterbury (Fig. 4 and Supplementary Fig. S11). The spatial pattern in the increasing NIR is similar for maize but to a lesser extent – 25–75 mm/year for SSP2-4.5 and 50–100 mm/year for SSP5-8.5 – and even less important for wheat with values mainly comprised between 0 and 50 in SSP4-8.5, and 25 and 75 for SSP5-8.5.

The results for SSP1-2.6 and SSP3-7.0 differ from those described for the other two SSPs with a projected decrease for most of the regions (Supplementary Figs. S9-S10). Indeed, while SSP2-4.5 and SSP5-8.5 project an increase in the NIR for the entire country and all the crops and periods, a *robust* decrease in the NIR is projected under SSP1-2.6 and SSP3-7.0 on the coast of NZ and in the Northland. *No changes or no robust signals* are projected elsewhere under SSP1-2.6, except a small patch in the centre of the North Island from the mid-term with a 0–50 mm/year increase. Under SSP3-7.0, the centre of both islands shows *robust signals* with an increase mainly ranging from 0 to 50 in the mid-term for all the crops, and 25 to 75 (maize and wheat) or 100 (apple and cherry) in the long-term.

## Discussion

### Agroclimatic suitability & net irrigation requirements

The historical land suitability results are consistent with previous studies, with a similar spatial suitability distribution pattern and intensity. For example, Vetharaniam et al.^13,14^ also found higher suitability for the North Island, the north of the South Island and the Canterbury regions for apple and results similar to our findings for cherry, although showing a lower suitability in Northland and higher in the south of the South Island. Regarding wheat and maize, the *viable* suitability observed in this study on the North Island, Marlborough and Canterbury matches the best suitability classes of previous studies^15,16^. The historical suitability spatial distribution is also consistent with current crop production, where regions producing the most for each crop present the highest suitability values over the historical period crop^38^. However, although current production crops are very localised, historical suitability results show that the suitability area extends far beyond current production boundaries and highlights the growing potential of each crop studied.

Because no water requirement criterion were considered in our LSA, suitability values are valid assuming that NIR are satisfied, and LSA results should be interpreted alongside NIR. However, the lack of information about irrigation rates in NZ over the historical period makes it difficult to validate NIR results. Oumarou Abdoulaye et al.^35^ suggested the method used may overestimate the NIR, but the magnitude of the possible estimation cannot be verified. The CWR used for the calculation of the NIR depends on external parameters like the presence of active ground cover and frost for fruit trees. In this study, K_C_ values corresponding to a crop with an active ground cover and killing frost were used for apple and cherry^33^, also leading to NIR overestimation in areas that don’t match this description.

The projected changes in suitability in the future for apple and cherry also match the general trend obtained in previous studies^13,14^, although with small differences. For example, Vetharaniam et al.^13^ found an increase in cherry suitability in the long term under RCP8.5 for almost all of the South Island, while our study shows negative changes in the North of the South Island as well as in the Canterbury region (Supplementary Fig. S8). These differences can be explained by the different climate projection datasets. While Vetheraniam et al.^13^ used CMIP5 projections, the up-to-date CMIP6 was used in this study. The projected changes in the maize suitability reveal the positive impacts of climate change on the production of this crop. On the other hand, although an increase in *viable* and *excellent* suitability for wheat is projected in Canterbury in most cases, some parts currently producing wheat in the region are expected to experience a decrease in suitability, highlighting the need to consider adaptation options. There are no results to compare our findings with for maize and wheat because no study has investigated future suitability for these two crops in NZ before.

The increases in projected suitability in the future should be interpreted with caution, as they are associated with increasing NIR for all the crops (Fig. 4 and Supplementary Fig. S9-S14) – this is especially true for crops with already high water requirements like maize. In addition, the projected increase in NIR of crops is consistent with previous studies that assessed the NIR under climate change conditions. For example, Busschaert et al. estimated a 30% increase in NIR in summer in the long term for a general C3-type crop^39^. Another study focusing on maize also suggests increasing NIR in the future, with variations according to the GCM and climate scenario used^35^.

The identification of the limiting factors for each crop is out of the scope of this study; however, we can highlight the lack of *excellent* suitability for cherry, whereas its production is very important in Otago ^38^. The land suitability of crops is influenced by each criterion used in LSA, and a low suitability value for one criterion can significantly degrade the final suitability score. One of the criterion used in the LSA of cherry was the fruit cracking (see Supplementary Methods), but poor suitability values were modelled for the latter, given the low resolution of climate data. Although its weight was decreased compared to its original use by Vetharaniam et al.^13^, its influence on land suitability was enough to drop areas out of the *excellent* class for the historical and future periods. Using higher resolution climate data, like Vetharaniam et al.^13^ may help to reduce this issue.

### Uncertainty, robustness and limits

In climate science, multi-model ensembles (MME) are highly useful to quantify model uncertainty and can also increase confidence in projected results^37^. In this study, the choice of the GCMs was made based on the GCMs used by Gibson et al.^27^ to downscale CMIP6 climate data on NZ, balancing their regional performance over the historical period, model independence and rate of future warming. Thus, the absence of the AWI-CM-1-1MR, being not available in the NEX-GDDP-CMIP6 dataset, changes the balance in the MME and leads to different results that would have been obtained with the MME of the six models.

Although several land suitability analyses have been conducted under climate in NZ and worldwide^6,14,31,40–42^, none of them have assessed the climate uncertainty associated with the future suitability demonstrating the significance of the uncertainty and robustness assessments of projected suitability carried out as part of this study. This was enabled by the use of the MME approach combined with the assumption that future land suitability is mainly driven by changing climate conditions, and provides additional useful information compared to previous studies. Thus, projected changes associated with a *robust signal* can be interpreted with a good certainty, while areas with *no change or robust signal*, or *conflicting signals*, need to be interpreted with more caution.

The results also show that apple and cherry tend to present more robust suitability changes than maize and wheat (Figs. 2 and 3 and Supplementary Figs. S5-S8), where the phenology of the crop was considered in the calculation of the criteria indicators only for the first ones. This suggests that integrating crop phenology in the computation of indicators used in LSA may increase the robustness and provide more confidence in the results, especially given the importance of considering phenology when studying climate change on crops^43^. Therefore, integrating the phenology of crops for maize and wheat, and improving its modelling approach for apple and cherry, can provide better LSA results, as well as using higher resolution climate data.

Although this work addresses the gaps in LSA regarding the consideration of CWR, providing estimates of NIR, it does not address seasonality changes. Indeed, only annual NIR were used, whereas changing climate conditions are affecting the total annual precipitation, but also the seasonality and intensity of precipitation^37^. Small changes in annual NIR appearing easily manageable can be misinterpreted as they might hide important seasonal variations strongly constraining crop production. Thus, although our findings suggest only small increases in NIR in the future for the four crops studied, more work is needed to simulate the complexity of water cycle changes.

As already highlighted, the spatial resolution of climate data can affect LSA results, especially considering the topography of NZ. Indeed, low climate resolution can smooth local climate effects and thus, underestimate or overestimate suitability, especially in small pockets of specific microclimate. In NZ, those small specific areas are often used for high-value crop production, such as cherry in Otago, the wine valleys of Central Otago, Waipara, and the Marlborough regions, or the production of hops in the Tasman valley. These high-value crops, located in specific areas because of a particular climatic regime, are especially vulnerable to the effects of climate change.

While this work studies the impacts of climate through LSA, it’s important to note that some aspects of climate change that also affect crop development are difficult to include in this approach and are thus not considered. For example, the increasing atmospheric carbon dioxide (CO_2_) concentration, one of the main drivers of climate change, enhances water use efficiency^44^ and can mitigate the projected increasing NIR. Therefore, it is necessary to bear in mind that the scope of this work focuses on the agroclimatic suitability of crops and does not encompass a complete view of climate change impacts on agriculture.

## Conclusion

This study extends our knowledge on the impact of climate change on NZ agriculture by conducting land suitability analysis (LSA) for four of the main crops – apple, cherry, maize and wheat. A consistent method was used across all the crops to perform LSA, relying on CMIP6 climate data and computing the NIR associated with suitability values, filling some gaps in previous LSA performed in NZ.

The suitability results over the historical period are consistent with the current production regions but also suggest a growing potential of the crops with *viable* suitability extended beyond current production areas. In addition, results show a general trend to an increase in suitability in the future in the South Island of NZ for the four crops studied, although varying in intensity and extent depending on the period, the SSP and the crop. More contrasted results were obtained for the North Island with a projected decreasing suitability in the long term for a majority of the area, with some patches of positive changes for apple, cherry and wheat, while an increasing suitability is projected for maize. These changes are associated with an increase in NIR for all crops that will need to be managed in the future.

Our findings on the NIR and the robustness of future suitability are valuable new information to nuance suitability results that can appear promising when focusing only on suitability value. However, only the annual NIR were studied, not allowing for the identification of seasonal changes in NIR that can constrain the production. Moreover, the land suitability strongly depends on the criteria used for the LSA, their associated indicators and how they are computed (e.g., integrating phenological modelling or not), but also on the spatial resolution of input data. Importantly, this study does not account for other biotic or abiotic stressors, such as pests and diseases, which could further influence crop viability under climate change. Thus, it is necessary to bear in mind the limits surrounding this work and not over-interpret the results.

The findings of this study can be used to inform policymakers, stakeholders, or land managers about areas where the production of a given crop is threatened by changing climate conditions and where adaptation strategies will be required in the future. In the same way, these findings can help to identify areas showing promising growing potential in the future, where conducting regional or local research would be interesting to better understand the growing potential.

Extending LSA to other crops (e.g., kiwifruit, avocado, winegrape…) is necessary to provide a more comprehensive overview of the impacts of climate change on NZ agriculture and will also enable more advanced study by integrating multi-crop analysis to better catch the complexity and diversity of agricultural production. Additionally, while this study focuses on NZ, the framework and methodology developed and used can readily be adapted and applied to other regions or countries.

## Electronic Supplementary Material

Below is the link to the electronic supplementary material.

Supplementary Material 1

## Data Availability

The code used for this study is accessible with the following link: https://doi.org/10.5281/zenodo.15768099. The generated datasets are available from the corresponding author on request. The LSAPy Python package on which the study relied is available here: https://doi.org/10.5281/zenodo.15015110.
