# Supplementary material for: Impacts of climate change on land suitability of key crops in New Zealand
Source: Sci Rep. 2026 Apr 22;16:18760. doi: 10.1038/s41598-026-49178-8 (PMC13273053; doi:10.1038/s41598-026-49178-8)
Supplement: Supplementary file 1 — Supplementary Material 1 [file 41598_2026_49178_MOESM1_ESM.pdf]

Impacts of Climate Change on Land Suitability of Key Crops in New Zealand

Baptiste Hamon, Hervé Quénol, Clémence Vannier, Thomas Cochrane

Supplementary Methods

Study Site

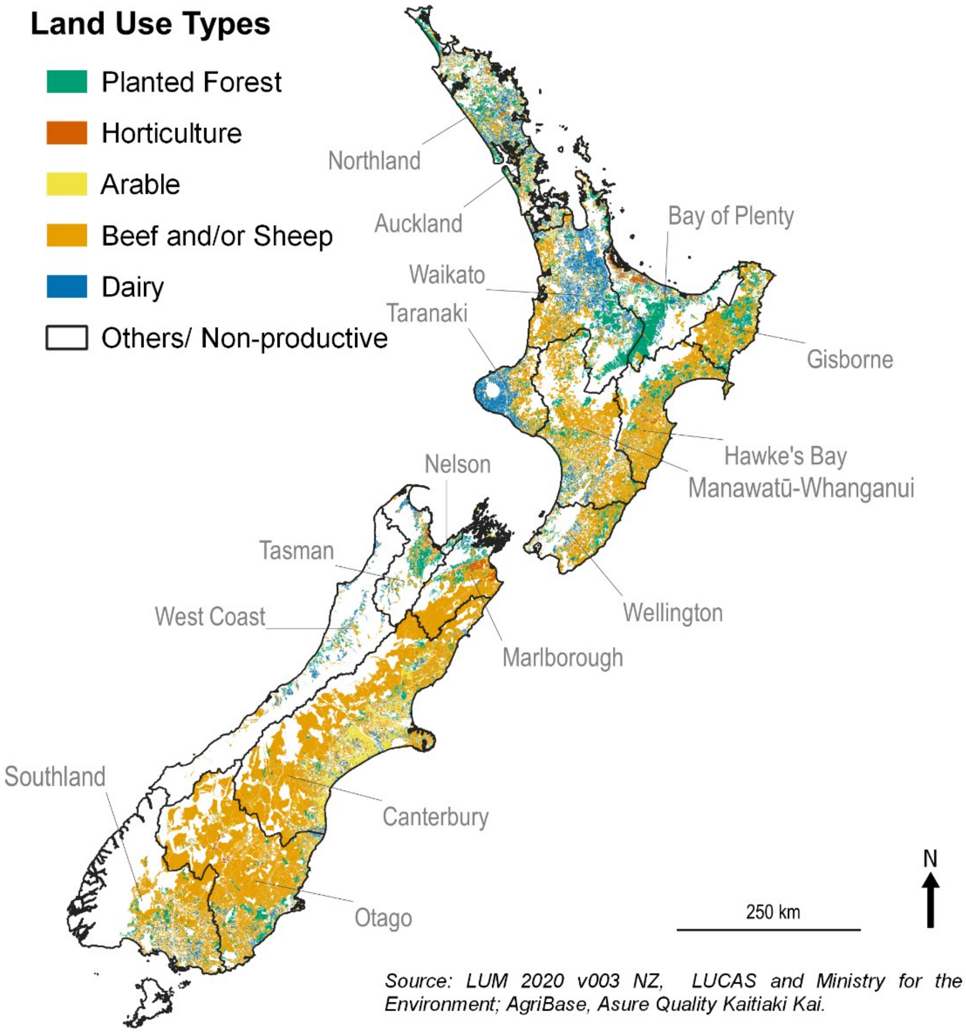

Figure S1 | New Zealand Agriculture Land Use Types. *Represented are the location of the five main agricultural sectors and the borders of administrative regions. The arable sector is mainly located in Canterbury while horticulture production is divided between the regions of Bay of Plenty, Hawke's Bay, Wellington, Marlborough, Tasman and Otago. The North Island contains the majority of the dairy industry, and the meat production is spread across the country — map generated with QGIS 3.16 (https://qgis.org).*

LSA Modelling

Membership Functions

Several equations are implemented in LSAPy to build the membership function. Those use in this study come from Vetharaniam *et al.*[1]:

$$y = \frac{1}{1 + e^{(-a(x-b)}} \tag{S1}$$

$$y = \frac{e^{\left(a(x-b)\right)}}{1 + e^{\left(a(x-b)\right)}} \tag{S2}$$

$$y = \frac{1}{1 + e^{\left(a(\sqrt{x}-\sqrt{b})\right)}} \tag{S3}$$

Apple LSA

***Table S1 | Apple LSA Criteria**. The criteria used for Apple's LSA are listed with their associated indicator, weight and function or rule applied to compute the criteria's suitability.*

Criteria	Indicator	Weight	Rule/ Function	Reference
Soil/Terrain				
- Potential Rooting Depth		1	Eq. S3 (a=-9.769, b=0.44)	
- Drainage Class		2	VP=0; P=0.3; I=0.6; MW=1; W=1	
- Land Use Capability Class		1	1=1; 2=0.95; 3=0.9; 4=0.8; 5=0.65; 6=05; 7=0.05; 8=0	
- Slope		0.5	Eq. S2 (a=-0.73, b=19)	
Climate				
- Winter Chill	Richardson Chill Unit | 1-May to 31-Aug	1	Eq. S3 (a=-0.445, b=896)	Vetharaniam *et al.*[1,2]
- Frost Survival Rate during Growth	Daily Minimum Temperature | DFB-21 to 30-Apr	2	Eq. S2 (a=1, b=-3)	
- Temperature for Maturation	Growing Degree Days (degCd); Base 10degC; 01-Oct to 15-Apr	1	Eq. S3 (a=-0.554, b=797)	
- Fruit Size	Growing Degree Days (degCd); Base 10degC; DFB* to DFB+50	1	Eq. S3 (a=-961, b=119)	
- Sunburn Survival Rate	Daily Maximum Temperature | 01-Oct to 30-Apr	0.5	Eq. S1 (a=-0.52, b=37.5)	

*DFB: Day of Full Bloom

The apple LSA considered phenological stages that makes the computation of the indicators more complex. The day of full bloom was used as start or end of some indicator's computation period and was calculated as:

$$DFB = 367 - 5.5 \times Tx \tag{S4}$$

where DFB is the day of full bloom in days since the 1st January, and Tx the mean maximum temperature over August and September.

The frost survival rate (FSR) during growth was calculated in two steps. First, the daily FSR was calculated on the minimum temperature with the Equation S2 and a=1 and b=-3 as parameters from 21 days before the DFB to the 30th April. To consider the variability in the timing of budbreak and the fruit harvesting, the losses (i.e., 1 - FSR) at the start and the end of the growing season

were down-weighted from 0 at the beginning of the frost period to 1 on day 8 for the start and from 1 on 1st April to 0 on 1st May. Then, the annual FSR was computed as the product of the daily FSR:

$$FSR = \prod 1 - (1 - FSR_i) \times w_i \qquad (S5)$$

where FSR and FSR_i are respectively the annual and daily survival rate and w_i the daily weight. The annual FSR was used as indicators and no additional suitability computation was made with LSAPy.

The same procedure was used to compute the sunburn survival rate (SSR), down-weighting only the end of the growing season.

Cherry LSA

***Table S2 | Cherry LSA Criteria**. The criteria used for Cherry's LSA are listed with their associated indicator, weight and function or rule applied to compute the criteria's suitability.*

Criteria	Indicator	Weight	Rule/ Function	Reference
Soil/Terrain				
- Potential Rooting Depth		1	Eq. S3 (a=-12.43, b=0.60)	
- Drainage Class		2	VP=0; P=0.2; I=0.4; MW=0.9; W=1	
- Land Use Capability Class		1	1=1; 2=0.95; 3=0.9; 4=0.8; 5=0.65; 6=05; 7=0.05; 8=0	
- Slope		0.5	Eq. S1 (a=-0.443, b=15)	
Climate				Vetharaniam *et al.*[1,2]
- Winter Chill	Richardson Chill Unit | 1-Jun to 31-Aug	1	Eq. S3 (a=-0.438, b=895)	
- Frost and Cold	Daily Minimum Temperature | Open cluster to maturity<br>Number of days from budbreak to 31st Dec	1	Eq. S2 (a=1.099, b=-3.4)<br>Eq. S2 (a=0.275, b=-77)	
- Temperature for Maturation	Growing Degree Days (degCd); Base 4.5degC; Budbreak to 30-Apr	2	Eq. S3 (a=-0.881, b=839)	
- Fruit Cracking	Daily Relative Humidity | 01-Nov to 30-Apr	0.25	Eq. S3(a=2.146, b=110.5)	

As for apple, the cherry's LSA considered the phenological stages of the crops. The budbreak was computed based on the cumulative sum of temperature above 4.5°C from 15 July to 30 April. The daily probability that the budbreak has occurred was computed using the Equation S2 with a=0.145 and b=174. The date of the budbreak was determined as the day when the probability is 0.5. The probability that the open cluster has occurred was calculated applying the Equation S3 with a=-3.36 and b=211 and the probability that ripening has occurred was calculated with the Equation S3 with a=-0.848 and b=839, but with daily temperatures cumulative sum weighted by the budbreak probability. The probability of being between the open cluster and the ripening dates was calculated by multiplying the probability of the open cluster and the ripening together.

For cherry, the frost and cold criteria was computed by combining the frost and the cold criteria to consider both frost risk during the growing season and too late budbreaking because of insufficient warm temperatures. The frost criteria corresponds to the frost survival rate and was computed as presented for apple but with daily weights corresponding to the probability of being in the growing season (open cluster to ripening probability). The cold criteria correspond to the time in days from budbreak to 31st December, assuming that budbreak occurring after 15 October (77 days before) will lead to a too short growing season. The Equation S2 with a=0.275 and b=77 was applied to compute the suitability of this criteria. The frost and cold criteria were then multiplied to create a unique criteria.

The fruit cracking criteria corresponds to the cracking survival rate and was computed in the same way as frost criteria with the same daily weights. In their previous study, Vetharaniam et al. [2] used a weight of 2 for fruit cracking criteria. However, computing with a coarser climate data resolution, the suitability of the cracking criteria is much smaller because the climate data does not catch the drier localised effect. As a result, the weight for this criteria was reduced to 0.25 in this study. The frost and cold, and cracking criteria were then used in LSAPy with no further modifications.

Maize LSA

Table S3 | Maize LSA Criteria*. The criteria used for maize's LSA are listed with their associated indicator, weight and function or rule applied to compute the criteria's suitability.*

Criteria	Indicator	Weight	Rule/ Function	Reference
Soil/Terrain				
- Potential Rooting Depth		1	Eq. S3 (a=-7.424, b=0.6)	
- Profile Available Water		1	Eq. S3 (a=-68.36, b=88.63)	
- Drainage Class		1	VP=0; P=0.1; I=0.5; MW=0.9; W=1	
- Slope		2	Eq. S3 (a=2.956, b=6.917)	
- Salinity	True if SAL >= 0.1% else False	0.25	True=0; False=1	Thomas et al.[3,4] Data Supermarket*
Climate				
- Rainfall Excess	Annual Total Precipitation	1	Eq. S3 (a=2.027, b=1400)	
- Frost during Growth	Frost (yrs/5yrs); Tmin < 0degC; 15-Sep to 15-Nov	1	Eq. S3 (a=-5.023, b=2.492)	
- Frost during Harvest	Frost (years in 5 years) | Tmin < -1oC | 15-Mar to 15-Apr	0.5	Eq. S3 (a=-2.709, b=2)	
- Temperature for Maturation	Growing Degree Days (degCd); Base 0degC; 15-Sep to 15-Dec	2	Eq. S3 (a=-1.821, b=1298)	

*https://landuseopportunities.nz/dataset/

Wheat LSA

Table S4 | Wheat LSA Criteria*. The criteria used for wheat's LSA are listed with their associated indicator, weight and function or rule applied to compute the criteria's suitability.*

Criteria	Indicator	Weight	Rule/ Function	Reference
Soil/Terrain				
- Potential Rooting Depth		1	Eq. S3 (a=-11.98, b=0.4588)	
- Profile Available Water		1	Eq. S3 (a=-68.36, b=88.63)	
- Drainage Class		1	VP=0; P=0.1; I=0.5; MW=0.9; W=1	
- Slope		2	Eq. S3 (a=2.956, b=6.917)	Thomas et al.[3,4] Data Supermarket*
- Salinity	True if SAL >= 0.1% else False	0.25	True=0; False=1	
Climate				
- Rainfall Excess	Annual Total Precipitation	1	Eq. S3 (a=0.8759, b=1248)	
- Frost during Growth	Frost (yrs/5yrs); Tmin < 0degC; 15-Sep to 15-Nov	1	Eq. S1 (a=-5.418, b=2.5)	

-	Frost during Winter	Frost (yrs/5yrs); Tmin < -10degC; 1-Jun to 31-Aug	0.5	Eq. S3 (a= 8.705, b= 2.998)
-	Temperature for Maturation	Growing Degree Days (degCd); Base 0degC; 15-Sep to 15-Dec	2	Eq. S3 (a=-1.821, b=1298)
-	Heat at Flowering	Heat wave (yrs/5yrs); > 3 seq days; Tmax > 25oC | 1-Nov to 31-Dec	1	Eq. S3 (a=-2.709, b=3)

*https://landuseopportunities.nz/dataset/

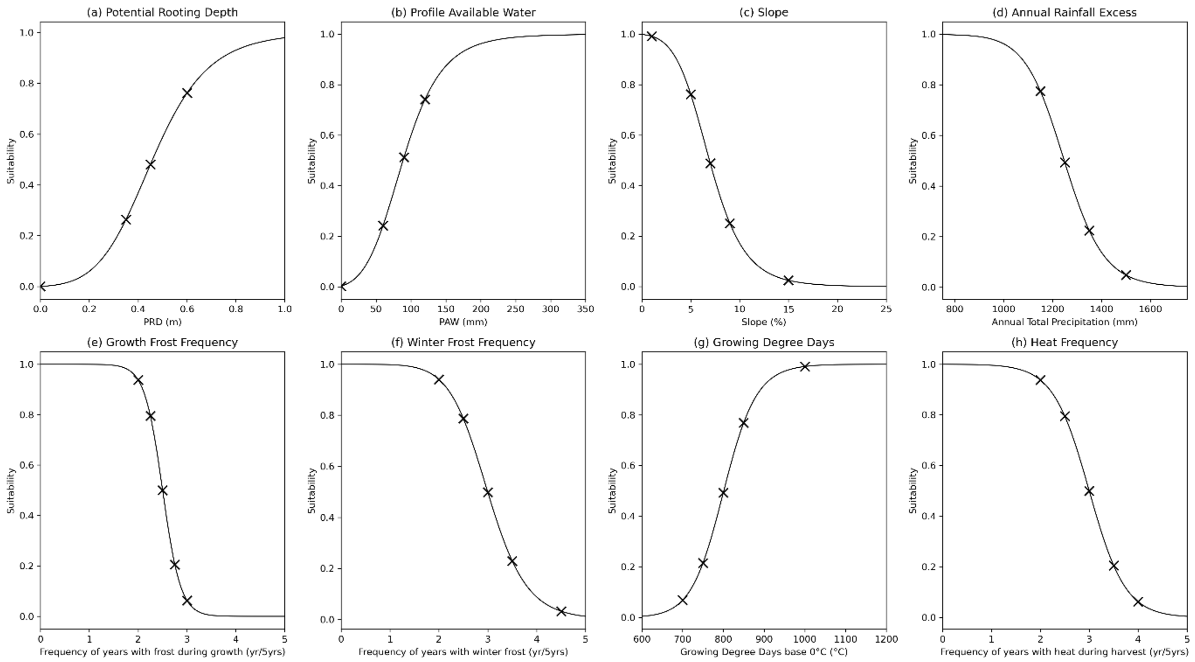

Figure S2 | Membership functions of continuous criteria used for LSA of wheat. *The black crosses correspond to values used to fit the functions.*

Crop Water Requirements

Table S5 | Crop parameters used to compute CWR. *The crop length of stages, K_C values and heights come from Allen et al [5]. The stage lengths correspond to high latitudes regions for apple and cherry, to Spain regions for maize, and to the Mediterranean and 35-45°L regions for winter and spring wheat. The K_C values for apple and cherry correspond to crop with active ground cover and killing frost, for winter wheat, they correspond to crop with frozen soils, and for maize, a mid-range late K_C value was used.*

Crop	Start date	Stage Length (days)				K_C			Height (m)
		Init.	Dev.	Mid	Late	Init.	Mid	Late	
Apple<br>Cherry	10-15	20	70	90	30	0.5	1.2	0.95	4
Maize	09-01	30	40	50	30	0.3	1.2	0.45	2
Wheat									
winter	05-15	30	140	40	30	0.4	1.15	0.25	1
spring	08-15	20	25	60	30	0.3	1.15	0.25	1

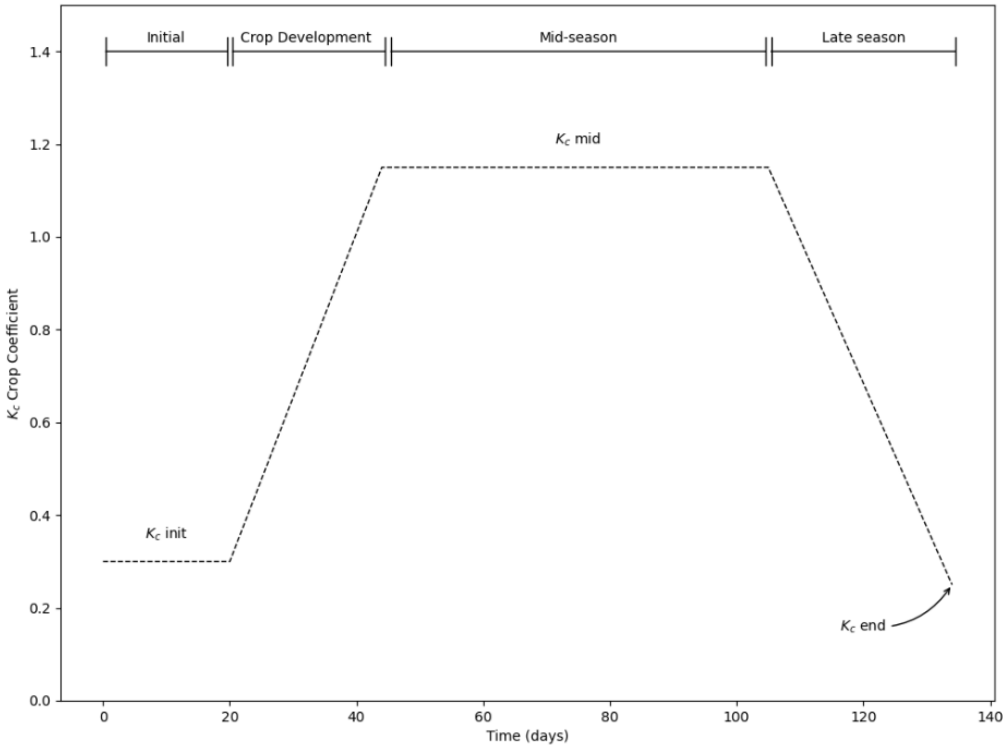

Figure S3 | Crop coefficient (Kc) curve for the different crop stages for wheat. *This curve represents the base curve and does not consider the adjusted Kc.*

Spatial Statistical Analysis

The conservation, natural forest, settlement and water areas were not considered as potential agricultural areas and were excluded for the spatial statistical analysis. The remaining total potential area for agriculture is 15,737,500ha – the current NZ agricultural area is 11,608,041ha (https://www.stats.govt.nz).

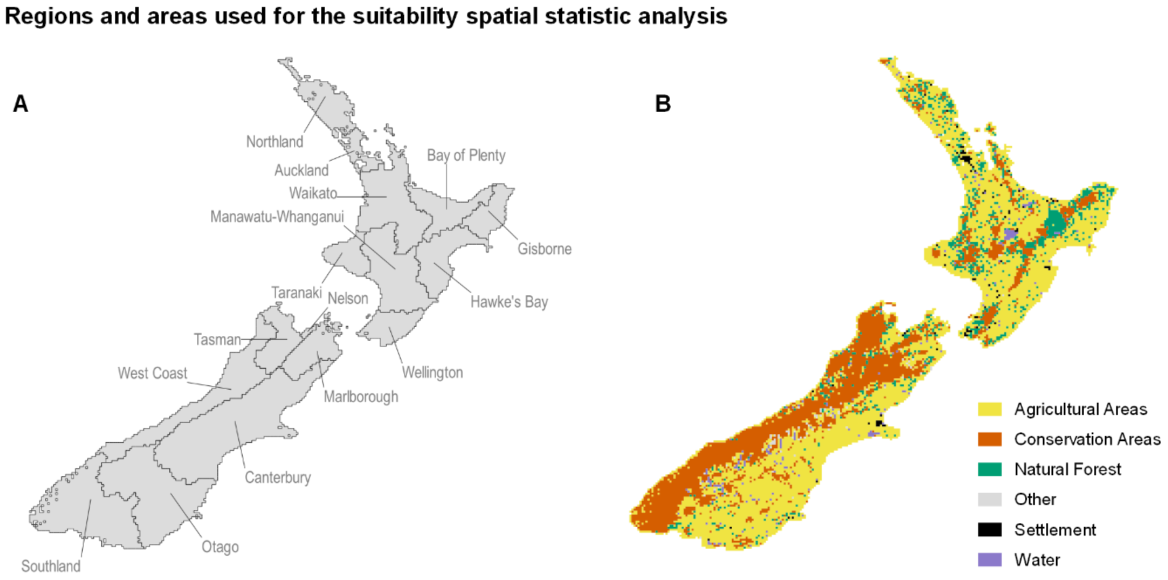

Figure S4 | Regions and Areas used for the Spatial Statistical Analysis. *The maps show the location of the administrative regions* ***(A)*** *and agricultural area* ***(B)*** *used for the regional statistics — maps generated with QGIS 3.16 (https://qgis.org).*

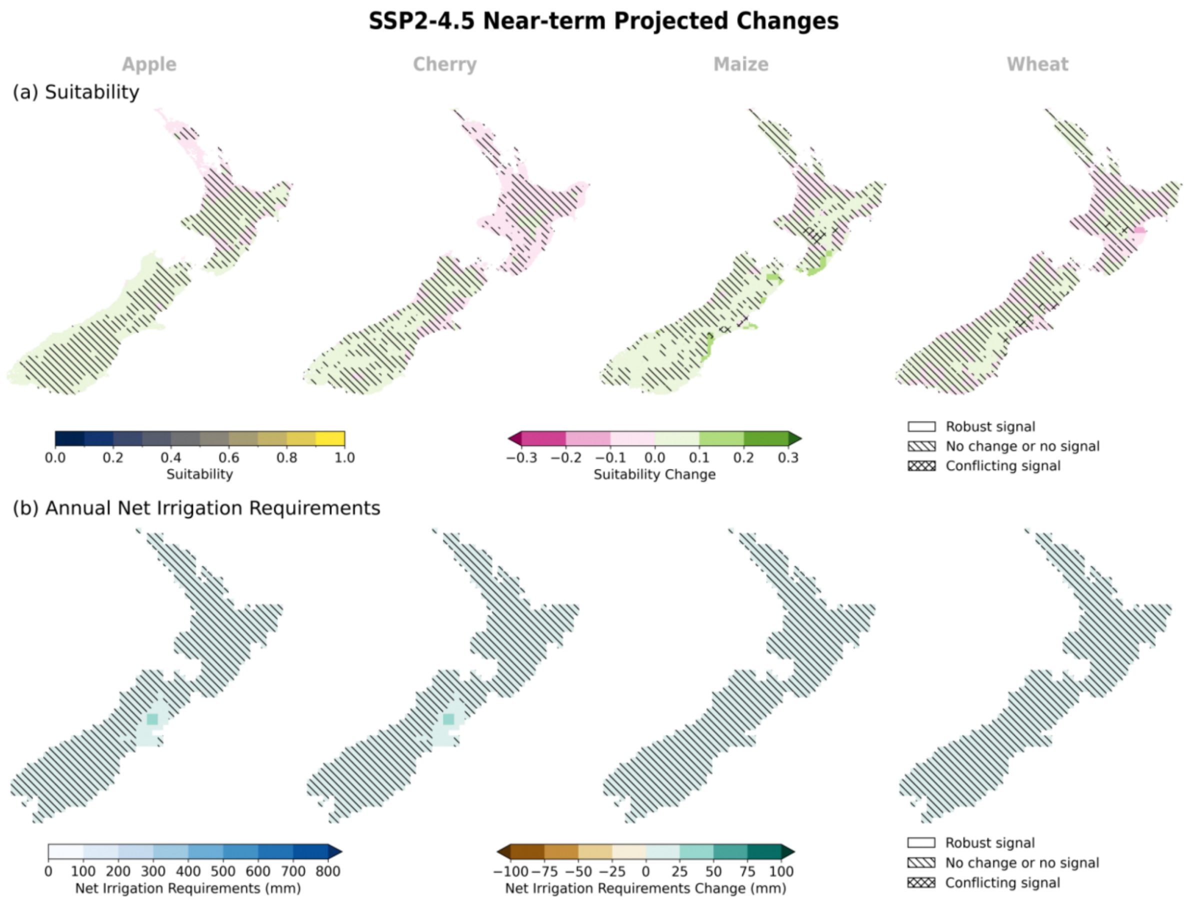

Figure S5 | Near-term crops suitability and annual crops Net Irrigation Requirements changes under SSP2-4.5. *Displayed are projected spatial patterns of multi-model (n=5) mean change in* ***(a)*** *crop suitability and* ***(b)*** *annual crop net irrigation requirements (NIR) with SSP2-4.5 in 2010-2039 relative to the 1980-2009 for apple, cherry, maize and wheat. The overlay represents the robustness[6]: no overlay indicates areas where the change is robust; diagonal lines indicate areas with no change or no robust significant change; crossed lines indicate areas of conflicting signals — maps generated in Python using matplotlib 3.9.2 (https://matplotlib.org).*

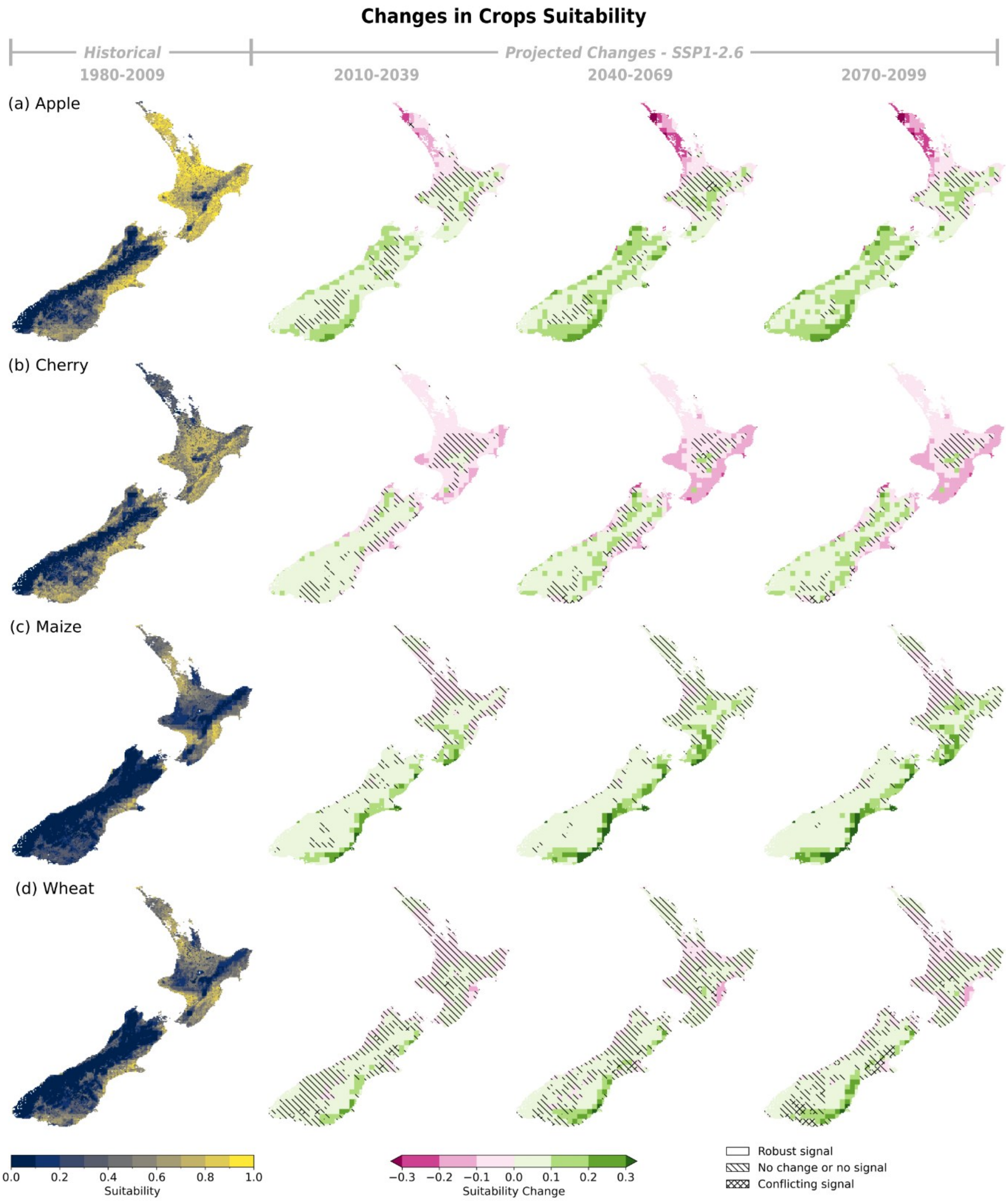

***Figure S6 | Historical crops suitability and near-, mid- and long-term changes.** Displayed are projected spatial patterns of multi-model (n=5) mean change in crop suitability with SSP1-2.6 in 2010-2039, 2040-2069 and 2070-2099 relative to the 1980-2009 historical suitability for **(a)** apple, **(b)** cherry, **(c)** maize and **(d)** wheat. The overlay represents the robustness[6]: no overlay indicates areas where the change is robust; diagonal lines indicate areas with no change or no robust significant change; crossed lines indicate areas of conflicting signals — maps generated in Python using matplotlib 3.9.2 (https://matplotlib.org).*

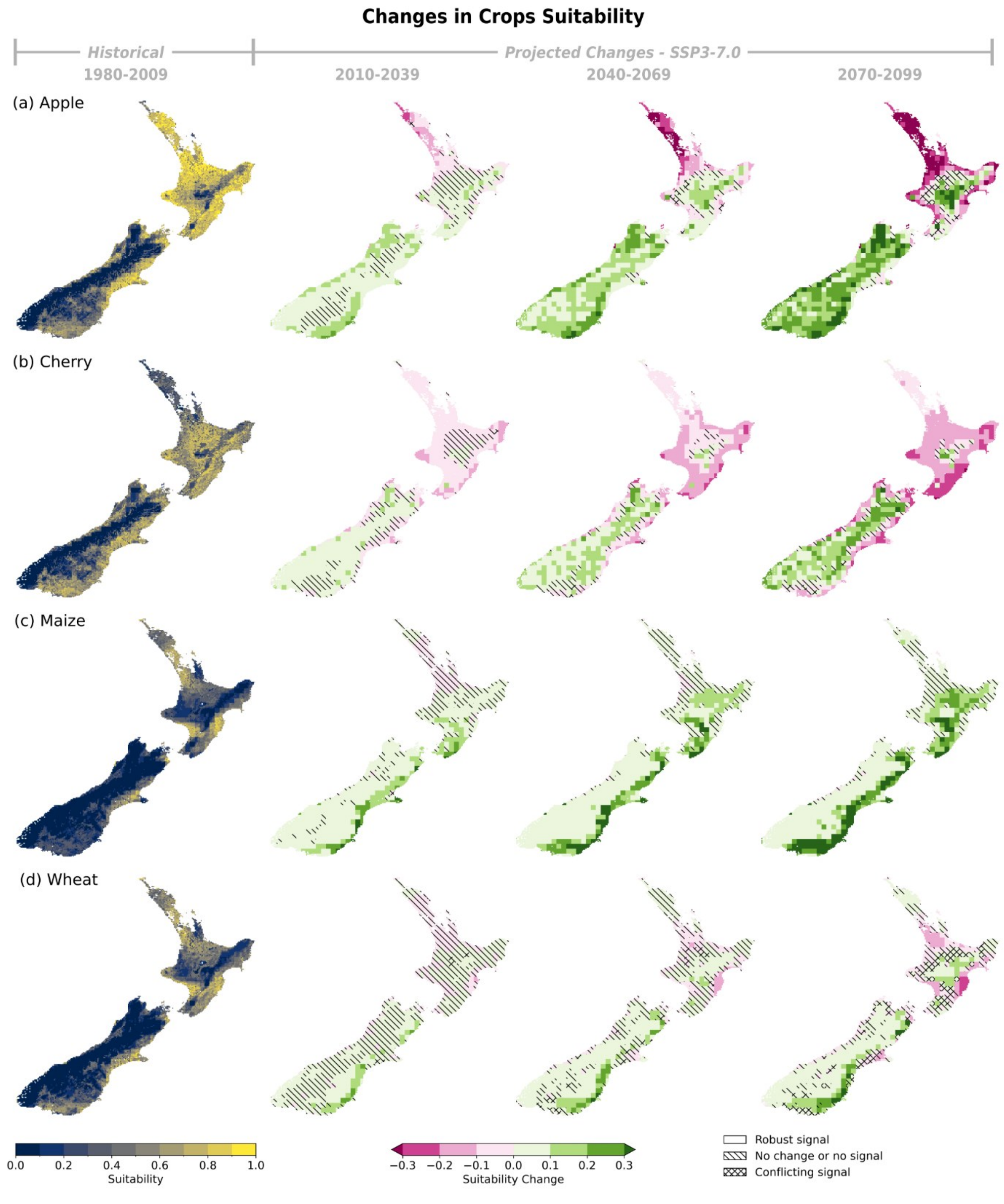

Figure S7 | Historical crops suitability and near-, mid- and long-term changes. *Displayed are projected spatial patterns of multi-model (n=5) mean change in crop suitability with SSP3-7.0 in 2010-2039, 2040-2069 and 2070-2099 relative to the 1980-2009 historical suitability for* ***(a)*** *apple,* ***(b)*** *cherry,* ***(c)*** *maize and* ***(d)*** *wheat. The overlay represents the robustness[6]: no overlay indicates areas where the change is robust; diagonal lines indicate areas with no change or no robust significant change; crossed lines indicate areas of conflicting signals — maps generated in Python using matplotlib 3.9.2 (https://matplotlib.org).*

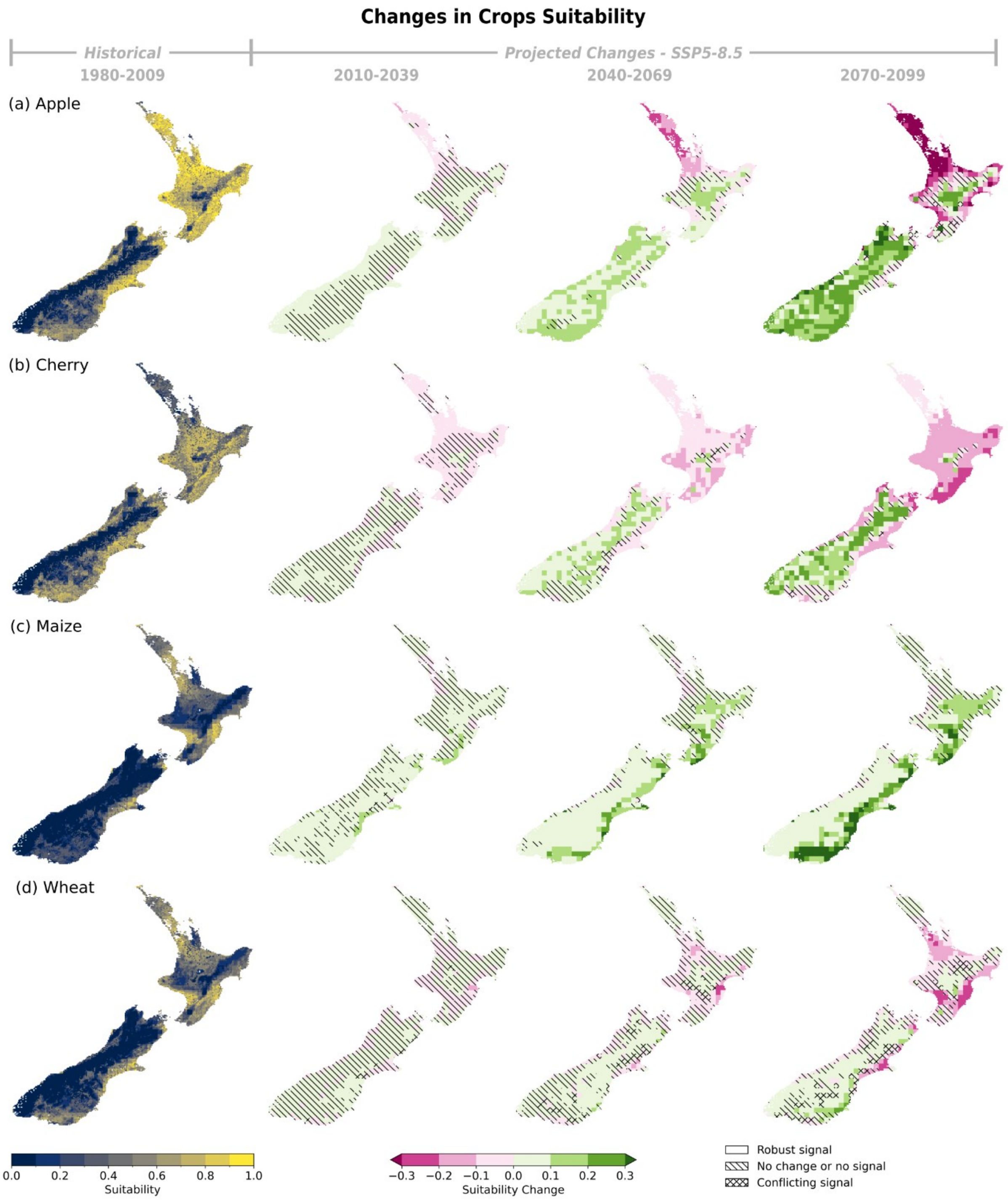

Figure S8 | Historical crops suitability and near-, mid- and long-term changes. *Displayed are projected spatial patterns of multi-model (n=5) mean change in crop suitability with SSP5-8.5 in 2010-2039, 2040-2069 and 2070-2099 relative to the 1980-2009 historical suitability for* ***(a)*** *apple,* ***(b)*** *cherry,* ***(c)*** *maize and* ***(d)*** *wheat. The overlay represents the robustness[6]: no overlay indicates areas where the change is robust; diagonal lines indicate areas with no change or no robust significant change; crossed lines indicate areas of conflicting signals — maps generated in Python using matplotlib 3.9.2 (https://matplotlib.org).*

***Table S6 | Historical and future long-term crops suitability areas (km²)**. Presented are the historical (1980-2009) and future long-term (2070-2099) multi-model (n=5) mean suitability areas in km² for SSP2-4.5 and SSP5-8.5 for apple, cherry, maize and wheat – in brackets are the corresponding multi-model standard deviations. The suitability areas are presented for each of the 16 New Zealand administrative regions and total of New Zealand. The suitability area is divided into two categories: viable **(V)** areas, where suitability ranges between 0.6 and 0.9, and excellent **(E)** areas, where suitability ranges between 0.9 and 1. For each region the potential agricultural area **(PAA)** in km² is given and statistics were computed only on this one, excluding areas unavailable for agriculture (see Spatial Statistical Analysis in Supplementary Methods).*

Region			Apple			Cherry			Maize			Wheat		
			Historical	Long term		Historical	Long term		Historical	Long term		Historical	Long term	
ID	PAA			SSP2-4.5	SSP5-8.5		SSP2-4.5	SSP5-8.5		SSP2-4.5	SSP5-8.5		SSP2-4.5	SSP5-8.5
NTL	7425	V	5200	2835 (1691)	565 (1040)	0	0	0	1850	2450 (892)	3145 (1304)	2900	3440 (654)	3270 (745)
		E	1500	115 (243)	0	0	0	0	0	0	0	50	50 (0)	35 (22)
AUK	2825	V	1950	920 (733)	195 (258)	250	5 (11)	0	2350	2385 (216)	2525 (110)	2425	2350(239)	1155 (778)
		E	700	55 (76)	0	0	0	0	0	0	0	0	0	0
WKO	17125	V	10300	12440 (1343)	8225 (3397)	10675	7050 (361)	4230 (827)	3125	4205 (2533)	4600 (2506)	5700	2765 (776)	575 (320)
		E	5250	1910 (731)	225 (140)	0	0	0	0	0	0	0	0	0
BOP	5850	V	3150	4205 (298)	3885 (896)	4275	3155 (286)	1825 (384)	0	350 (446)	475 (684)	1100	595 (214)	130 (143)
		E	2200	1305 (293)	110 (58)	0	0	0	0	0	0	0	0	0
GIS	6100	V	4125	4480 (356)	3745 (1263)	2800	1195 (527)	505 (217)	225	530 (401)	755 (442)	375	300 (128)	105 (33)
		E	1600	1430 (372)	410 (329)	0	0	0	0	0	0	0	0	0
HKB	10000	V	6300	7190 (504)	6850 (1683)	6550	4415 (797)	2170 (972)	4900	5475 (1173)	5965 (1025)	4750	1510 (612)	540 (175)
		E	2650	2210 (592)	540 (231)	0	0	0	125	95 (45)	125 (0)	50	0	0
TKI	4525	V	2625	2375 (352)	3005 (618)	3525	2525 (516)	1270 (810)	250	305 (110)	315 (191)	1700	1445 (504)	995 (809)
		E	1775	2075 (352)	535 (485)	0	0	0	0	0	0	25	5 (11)	0
MWT	16000	V	12825	12050 (740)	11380 (1429)	9575	7160 (180)	4285 (580)	4750	7670 (2729)	8890 (1715)	5700	4515 (1448)	1900 (627)
		E	1025	2480 (1220)	1015 (202)	0	0	0	0	25 (25)	70 (102)	225	35 (49)	0
WGN	5175	V	4550	3750 (388)	4270 (481)	3500	1635 (675)	550 (589)	525	3145 (706)	3610 (859)	1600	1965 (450)	1020 (891)
		E	450	1315 (401)	665 (604)	0	0	0	0	0	5 (11)	0	0	0
TAS	2700	V	1725	1945 (121)	2065 (106)	1575	1375 (94)	860 (57)	0	10 (14)	0	25	35 (38)	0
		E	100	425 (171)	410 (98)	0	0	0	0	0	0	0	0	0
MBH	4825	V	2600	2710 (118)	3250 (252)	2100	1990 (156)	1235 (208)	625	1265 (229)	1425 (465)	875	915 (155)	365 (290)
		E	125	645 (203)	285 (88)	0	0	0	75	145 (45)	155 (54)	50	20 (27)	0
NSN	200	V	125	175 (0)	180 (11)	100	80 (11)	35 (38)	0	0	0	0	0	0
		E	25	25 (0)	20 (11)	0	0	0	0	0	0	0	0	0
WTC	2200	V	1200	1295 (69)	1585 (171)	1150	1155 (124)	685 (128)	25	25 (0)	25 (0)	25	25 (0)	25 (0)
		E	0	320 (246)	195 (51)	0	0	0	0	0	0	0	0	0
CAN	32300	V	14825	17315 (2148)	21130 (3735)	15325	16790 (3615)	14080 (1973)	3525	9430 (4916)	13030 (4376)	5925	6145 (3868)	4360 (1165)
		E	1200	3390 (2353)	2340 (984)	0	0	0	0	735 (704)	1095 (648)	250	250 (143)	50 (73)
OTA	26300	V	3825	10015 (3935)	13190 (4257)	7225	10285 (3088)	9965 (2474)	0	2445 (2047)	6005 (3731)	1700	3465 (2022)	4590 (2341)
		E	0	615 (668)	1160 (1091)	0	0	0	0	75 (113)	230 (190)	50	205 (179)	85 (29)
STL	13750	V	4325	7340 (778)	8525 (1250)	6700	7705 (1267)	6705 (444)	0	2385 (2961)	3780 (3134)	2625	3025 (2534)	3100 (359)
		E	0	1300 (1821)	1790 (2164)	0	0	0	0	45 (101)	70 (98)	0	315 (446)	0
NZ	157375	V	79650	91040 (2997)	92045 (4073)	75325	66425 (4941)	48400 (2383)	22150	42075 (15350)	54545 (17843)	37425	32495 (9758)	22140 (2277)
		E	18600	19615 (7616)	9700 (3654)	0	0	0	200	1120 (960)	1750 (986)	700	900 (553)	170 (102)

Regions: Northland (NTL), Auckland (AUK), Waikato (WKO), Bay of Plenty (BOP), Gisborne (GIS), Hawke's Bay (HKB), Taranaki (TKI), Manawatū-Whanganui (MWT), Wellington (WGN), West Coast (WTC), Canterbury (CAN), Otago (OTA), Southland (STL), Tasman (TAS), Nelson (NSN), Marlborough (MBH), New Zealand (NZ).

Table S7 | Historical and mid-term crops suitability areas (km2). *Presented are the historical (1980-2009) and mid-term (2040-2069) multi-model (n=5) mean suitability areas in km2 for SSP1-2.6 and SSP3-7.0 for apple, cherry, maize and wheat – in brackets are the corresponding multi-model standard deviations. The suitability areas are presented for each of the 16 New Zealand administrative regions and total of New Zealand. The suitability area is divided into two categories: viable **(V)** areas, where suitability ranges between 0.6 and 0.9, and excellent **(E)** areas, where suitability ranges between 0.9 and 1. For each region the potential agricultural area **(PAA)** in km^2 is given and statistics were computed only on this one, excluding areas unavailable for agriculture (see Spatial Statistical Analysis in Supplementary Methods).*

Region			Apple			Cherry			Maize			Wheat		
			Historical	Mid term		Historical	Mid term		Historical	Mid term		Historical	Mid term	
ID	PAA			SSP1-2.6	SSP3-7.0		SSP1-2.6	SSP3-7.0		SSP1-2.6	SSP3-7.0		SSP1-2.6	SSP3-7.0
NTL	7425	V	5200	3415 (1237)	2115 (1064)	0	0	0	1850	2220 (645)	2175 (326)	2900	3230 (535)	3335 (365)
		E	1500	220 (309)	10 (22)	0	0	0	0	0	0	50	50	50 (0)
AUK	2825	V	1950	1230 (595)	585 (309)	250	35 (55)	0	2350	2410 (123)	2400 (64)	2425	2460 (84)	2425 (88)
		E	700	95 (41)	30 (54)	0	0	0	0	0	0	0	0	0
WKO	17125	V	10300	11370 (1145)	12095 (1245)	10675	7620 (371)	6945 (173)	3125	3755 (1033)	3935 (1478)	5700	5480 (1101)	4990 (1512)
		E	5250	3430 (1031)	2055 (1050)	0	0	0	0	0	0	0	0	0
BOP	5850	V	3150	3675 (552)	4085 (434)	4275	3485 (178)	3075 (88)	0	200 (358)	215 (316)	1100	1235 (494)	1245 (455)
		E	2200	1825 (653)	2275 (715)	0	0	0	0	0	0	0	0	0
GIS	6100	V	4125	4325 (188)	4555 (256)	2800	1110 (439)	715 (161)	225	340 (270)	410 (122)	375	335 (22)	315 (45)
		E	1600	1595 (196)	1130 (350)	0	0	0	0	0	0	0	0	0
HKB	10000	V	6300	6415 (297)	7120 (660)	6550	4120 (933)	3365 (661)	4900	5445 (502)	5610 (530)	4750	3380 (562)	2855 (1073)
		E	2650	2905 (480)	2310 (600)	0	0	0	125	125 (0)	125 (0)	50	15 (14)	10 (14)
TKI	4525	V	2625	2630 (427)	3140 (366)	3525	1860 (822)	1355 (805)	250	335 (58)	340 (82)	1700	1985 (427)	1855 (379)
		E	1775	1775 (472)	1120 (535)	0	0	0	0	0	0	25	30 (11)	10 (14)
MWT	16000	V	12825	11105 (1098)	11845 (850)	9575	7565 (608)	6890 (449)	4750	7945 (1744)	8495 (1925)	5700	6465 (1573)	5775 (758)
		E	1025	3035 (1560)	2830 (1027)	0	0	0	0	45 (27)	35 (14)	225	325 (129)	20 (11)
WGN	5175	V	4550	3570 (330)	3705 (432)	3500	1400 (752)	990 (562)	525	3455 (500)	3720 (634)	1600	2980 (259)	2570 (312)
		E	450	1490 (340)	1350 (437)	0	0	0	0	0	0	0	0	0
TAS	2700	V	1725	1830 (155)	1950 (73)	1575	1405 (139)	1340 (158)	0	5 (11)	5 (11)	25	45 (37)	50 (47)
		E	100	425 (176)	470 (179)	0	0	0	0	0	0	0	0	0
MBH	4825	V	2600	2645 (84)	2705 (65)	2100	1935 (104)	1765 (150)	625	1265 (204)	1400 (146)	875	1030 (219)	1070 (76)
		E	125	660 (225)	765 (171)	0	0	0	75	135 (38)	140 (34)	50	50 (0)	40 (22)
NSN	200	V	125	160 (22)	170 (11)	100	80 (11)	75 (0)	0	0	0	0	0	0
		E	25	25 (0)	25 (0)	0	0	0	0	0	0	0	0	0
WTC	2200	V	1200	1240 (52)	1240 (70)	1150	1055 (132)	980 (84)	25	25 (0)	25 (0)	25	25 (0)	25 (0)
		E	0	375 (207)	445 (145)	0	0	0	0	0	0	0	0	0
CAN	32300	V	14825	15590 (1204)	16795 (1388)	15325	16040 (2503)	15285 (2279)	3525	10615 (4087)	12055 (3604)	5925	8585 (4152)	8995 (3647)
		E	1200	4780 (2510)	4480 (2306)	0	5 (11)	0	0	970 (500)	1040 (508)	250	1490 (831)	1075 (267)
OTA	26300	V	3825	9740 (3039)	11020 (2674)	7225	10165 (1939)	10000 (1760)	0	3170 (1907)	5010 (1638)	1700	5320 (2099)	5800 (1933)
		E	0	1060 (751)	1495 (1061)	0	0	0	0	125 (141)	180 (177)	50	490 (339)	425 (193)
STL	13750	V	4325	6685 (702)	7240 (310)	6700	7505 (1046)	6960 (211)	0	1865 (2603)	2650 (3186)	2625	3470 (2544)	3385 (2261)
		E	0	1235 (1719)	1630 (1961)	0	0	0	0	0	35 (78)	0	425 (508)	360 (455)
NZ	157375	V	79650	85625 (2020)	90365 (2610)	75325	65380 (3773)	60435 (3587)	22150	43045 (12950)	48445 (12720)	37425	46025 (12109)	44690 (7677)
		E	18600	24930 (8449)	22050 (7718)	0	5 (11)	0	200	1400 (665)	1555 (768)	700	2875 (1631)	2065 (760)

Regions: Northland (NTL), Auckland (AUK), Waikato (WKO), Bay of Plenty (BOP), Gisborne (GIS), Hawke's Bay (HKB), Taranaki (TKI), Manawatū-Whanganui (MWT), Wellington (WGN), West Coast (WTC), Canterbury (CAN), Otago (OTA), Southland (STL), Tasman (TAS), Nelson (NSN), Marlborough (MBH), New Zealand (NZ).

Table S8 | Historical and long-term crops suitability areas (km2). *Presented are the historical (1980-2009) and long-term (2070-2099) multi-model (n=5) mean suitability areas in km2 for SSP1-2.6 and SSP3-7.0 for apple, cherry, maize and wheat – in brackets are the corresponding multi-model standard deviations. The suitability areas are presented for each of the 16 New Zealand administrative regions and total of New Zealand. The suitability area is divided into two categories: viable* ***(V)*** *areas, where suitability ranges between 0.6 and 0.9, and excellent* ***(E)*** *areas, where suitability ranges between 0.9 and 1. For each region the potential agricultural area* ***(PAA)*** *in km^2 is given and statistics were computed only on this one, excluding areas unavailable for agriculture (see Spatial Statistical Analysis in Supplementary Methods).*

Region			Apple			Cherry			Maize			Wheat		
			Historical	Long term		Historical	Long term		Historical	Long term		Historical	Long term	
ID	PAA			SSP1-2.6	SSP3-7.0		SSP1-2.6	SSP3-7.0		SSP1-2.6	SSP3-7.0		SSP1-2.6	SSP3-7.0
NTL	7425	V	5200	3290 (1130)	800 (1257)	0	0	0	1850	1850 (337)	3310 (1102)	2900	2960 (439)	3930 (336)
		E	1500	145 (249)	0	0	0	0	0	5575 (337)	0	50	50 (0)	50 (0)
AUK	2825	V	1950	1060 (435)	215 (207)	250	15 (22)	0	2350	2410 (123)	2550 (124)	2425	2390 (99)	1975 (613)
		E	700	100 (56)	0	0	0	0	0	0	0	0	0	0
WKO	17125	V	10300	11465 (1699)	8645 (2197)	10675	7620 (371)	5265 (571)	3125	3270 (1134)	6025 (1840)	5700	5220 (1745)	3105 (1812)
		E	5250	3545 (1573)	1215 (753)	0	0	0	0	0	0	0	0	0
BOP	5850	V	3150	3555 (644)	3855 (220)	4275	3530 (149)	2065 (261)	0	155 (250)	645 (404)	1100	1105 (589)	775 (418)
		E	2200	2025 (630)	805 (563)	0	0	0	0	0	0	0	0	0
GIS	6100	V	4125	4395 (321)	3460 (921)	2800	1100 (378)	455 (142)	225	340 (270)	660 (195)	375	330 (65)	195 (111)
		E	1600	1530 (321)	555 (284)	0	0	0	0	0	0	0	0	0
HKB	10000	V	6300	6310 (637)	6465 (1313)	6550	4055 (911)	2150 (683)	4900	5260 (818)	6105 (478)	4750	3390 (873)	1190 (569)
		E	2650	3050 (616)	1200 (813)	0	0	0	125	125 (0)	125 (0)	50	5 (11)	0
TKI	4525	V	2625	2570 (493)	2635 (927)	3525	1860 (822)	620 (568)	250	295 (51)	375 (146)	1700	1755 (440)	1455 (646)
		E	1775	1835 (564)	390 (307)	0	0	0	0	0	0	25	25 (0)	0
MWT	16000	V	12825	11145 (1358)	10910 (1390)	9575	7610 (654)	5100 (808)	4750	7585 (1884)	9930 (1026)	5700	5970 (1798)	4880 (2113)
		E	1025	3240 (1720)	2265 (1063)	0	0	0	0	40 (29)	70 (74)	225	160 (123)	5 (11)
WGN	5175	V	4550	3585 (408)	4015 (526)	3500	1400 (752)	205 (143)	525	3340 (673)	3840 (619)	1600	2550 (581)	1915 (1225)
		E	450	1480 (418)	740 (706)	0	0	0	0	0	0	0	0	0
TAS	2700	V	1725	1820 (122)	2035 (146)	1575	1405 (139)	930 (203)	0	0	15 (22)	25	65 (49)	25 (43)
		E	100	430 (179)	515 (162)	0	0	0	0	0	0	0	0	0
MBH	4825	V	2600	2645 (62)	3130 (266)	2100	1945 (114)	1285 (1710)	625	1255 (187)	1625 (288)	875	1065 (183)	785 (374)
		E	125	685 (223)	515 (143)	0	0	0	75	130 (41)	175 (47)	50	50 (0)	10 (22)
NSN	200	V	125	165 (22)	175 (0)	100	80 (11)	30 (33)	0	0	0	0	0	0
		E	25	25 (0)	25 (0)	0	0	0	0	0	0	0	0	0
WTC	2200	V	1200	1245 (60)	1395 (135)	1150	1055 (91)	765 (205)	25	25 (0)	25 (0)	25	25 (0)	25 (0)
		E	0	365 (218)	455 (120)	0	0	0	0	0	0	0	0	0
CAN	32300	V	14825	15850 (1254)	20395 (2579)	15325	15920 (1851)	13875 (1777)	3525	106130 (4257)	14785 (2761)	5925	8585 (4276)	8230 (1654)
		E	1200	4820 (2477)	4065 (1351)	0	10 (14)	0	0	850 (590)	134 (429)	250	1430 (1033)	175 (129)
OTA	26300	V	3825	10390 (2675)	13330 (2878)	7225	10255 (1326)	9465 (1941)	0	4075 (1820)	7280 (2555)	1700	5895 (2034)	6950 (1894)
		E	0	1150 (783)	2180 (1230)	0	0	0	0	130 (129)	325 (159)	50	550 (389)	330 (207)
STL	13750	V	4325	7185 (443)	7750 (273)	6700	7650 (1046)	6960 (211)	0	1880 (2668)	4420 (2216)	2625	4045 (2111)	4645 (2176)
		E	0	1260 (1690)	2480 (1999)	0	0	0	0	0	170 (292)	0	375 (449)	75 (71)
NZ	157375	V	79650	86675 (2704)	88890 (3104)	75325	65730 (3190)	49170 (4089)	22150	42240 (13528)	61590 (10713)	37425	45350 (13031)	40080 (5320)
		E	18600	25685 (9657)	17405 (5443)	0	10 (14)	0	200	1275 (771)	2210 (914)	700	2645 (1833)	645 (320)

Regions: Northland (NTL), Auckland (AUK), Waikato (WKO), Bay of Plenty (BOP), Gisborne (GIS), Hawke's Bay (HKB), Taranaki (TKI), Manawatū-Whanganui (MWT), Wellington (WGN), West Coast (WTC), Canterbury (CAN), Otago (OTA), Southland (STL), Tasman (TAS), Nelson (NSN), Marlborough (MBH), New Zealand (NZ).

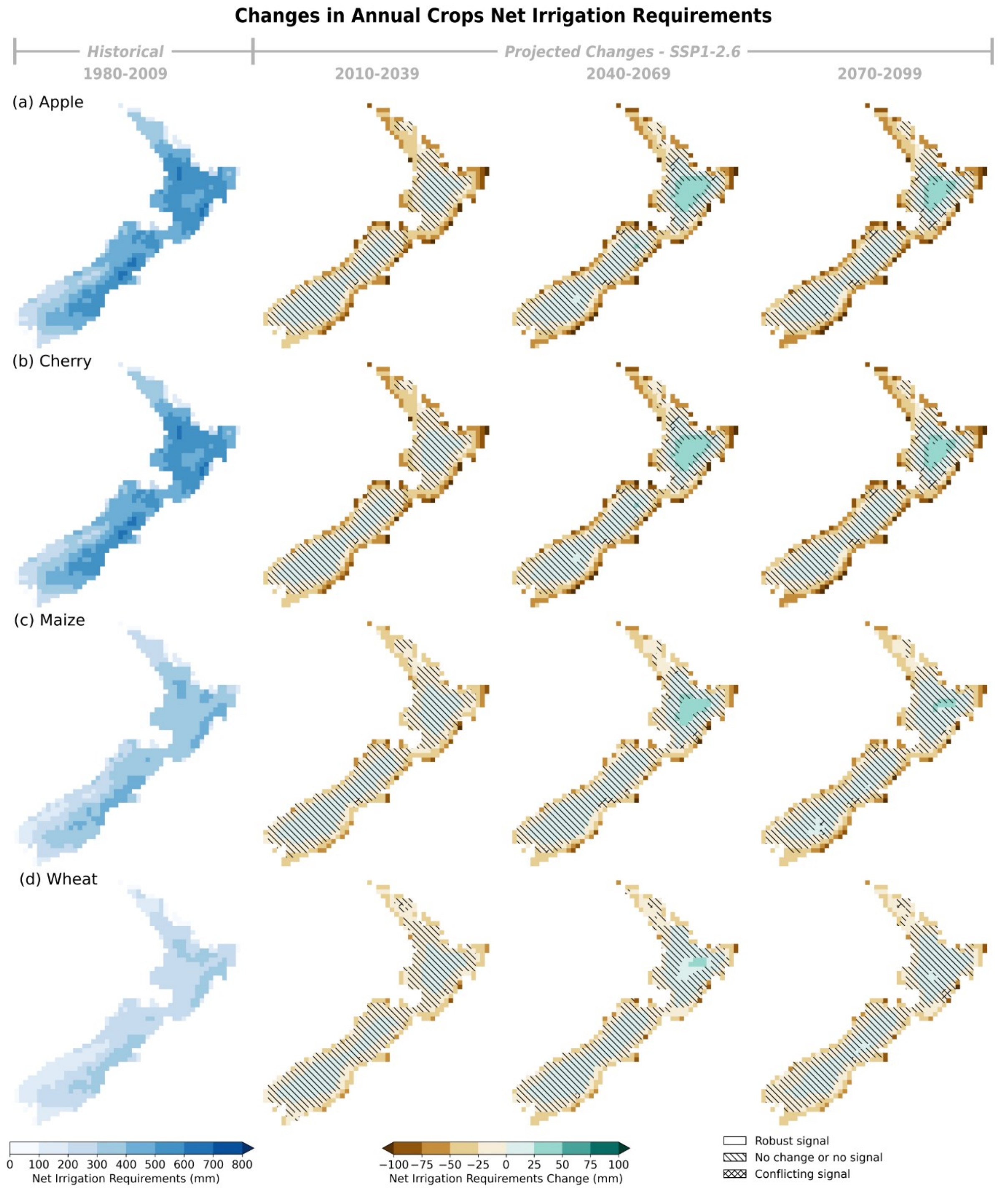

***Figure S9 | Historic annual crops net irrigation requirements (NIR) and near-, mid- and long-term changes**. Displayed are projected spatial patterns of multi-model (n=5) mean change in annual crop net irrigation requirements (NIR) with SSP1-2.6 in 2010-2039, 2040-2069 and 2070-2099 relative to the 1980-2009 historic NIR for **(a)** apple, **(b)** cherry, **(c)** maize and **(d)** wheat. The overlay represents the robustness[6]: no overlay indicates areas where the change is robust; diagonal lines indicate areas with no change or no robust significant change; crossed lines indicate areas of conflicting signals — maps generated in Python using matplotlib 3.9.2 (https://matplotlib.org).*

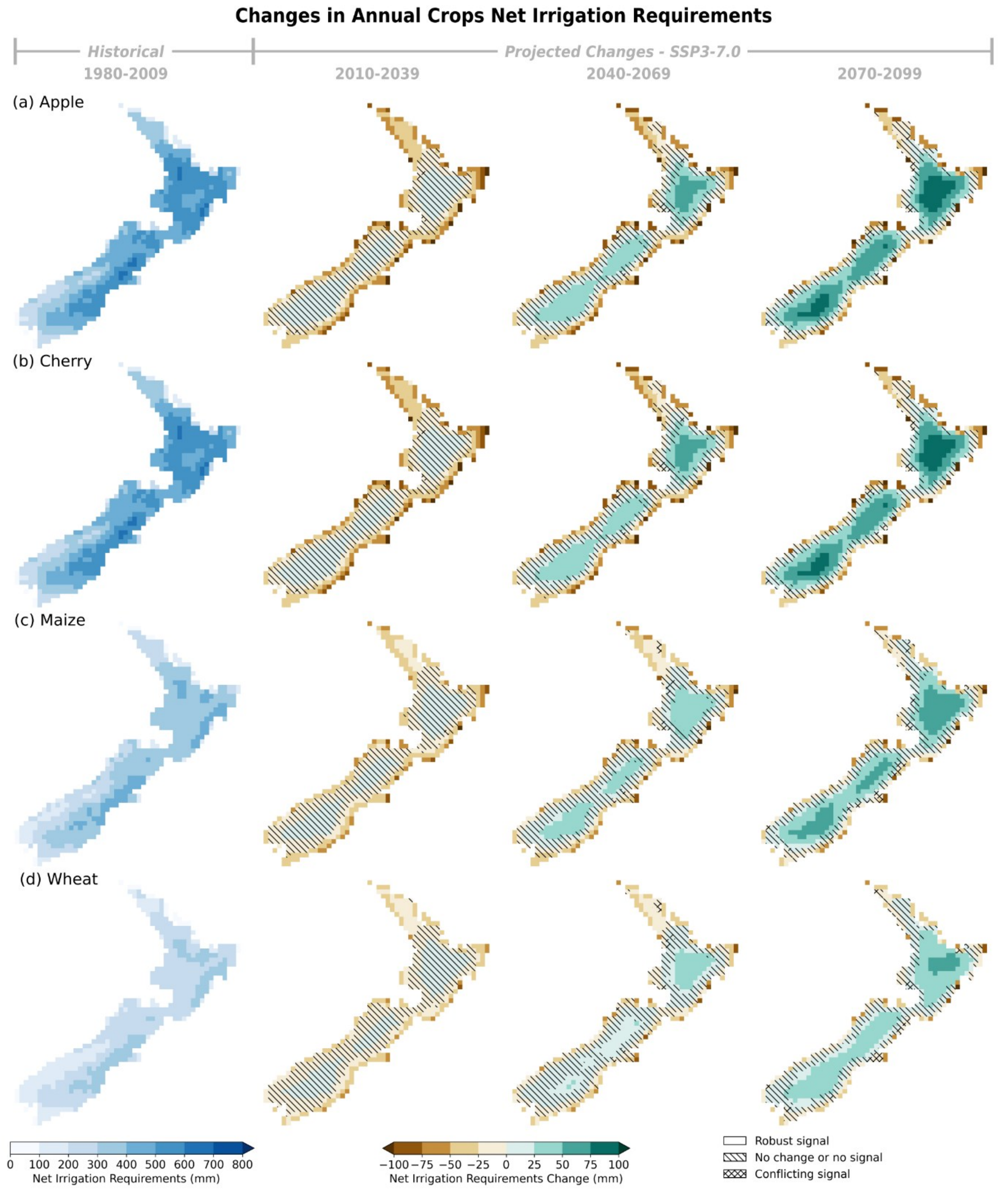

Figure S10 | Historic annual crops net irrigation requirements (NIR) and near-, mid- and long-term changes. *Displayed are projected spatial patterns of multi-model (n=5) mean change in annual crop net irrigation requirements (NIR) with SSP3-7.0 in 2010-2039, 2040-2069 and 2070-2099 relative to the 1980-2009 historic NIR for* ***(a)*** *apple,* ***(b)*** *cherry,* ***(c)*** *maize and* ***(d)*** *wheat. The overlay represents the robustness[6]: no overlay indicates areas where the change is robust; diagonal lines indicate areas with no change or no robust significant change; crossed lines indicate areas of conflicting signals — maps generated in Python using matplotlib 3.9.2 (https://matplotlib.org).*

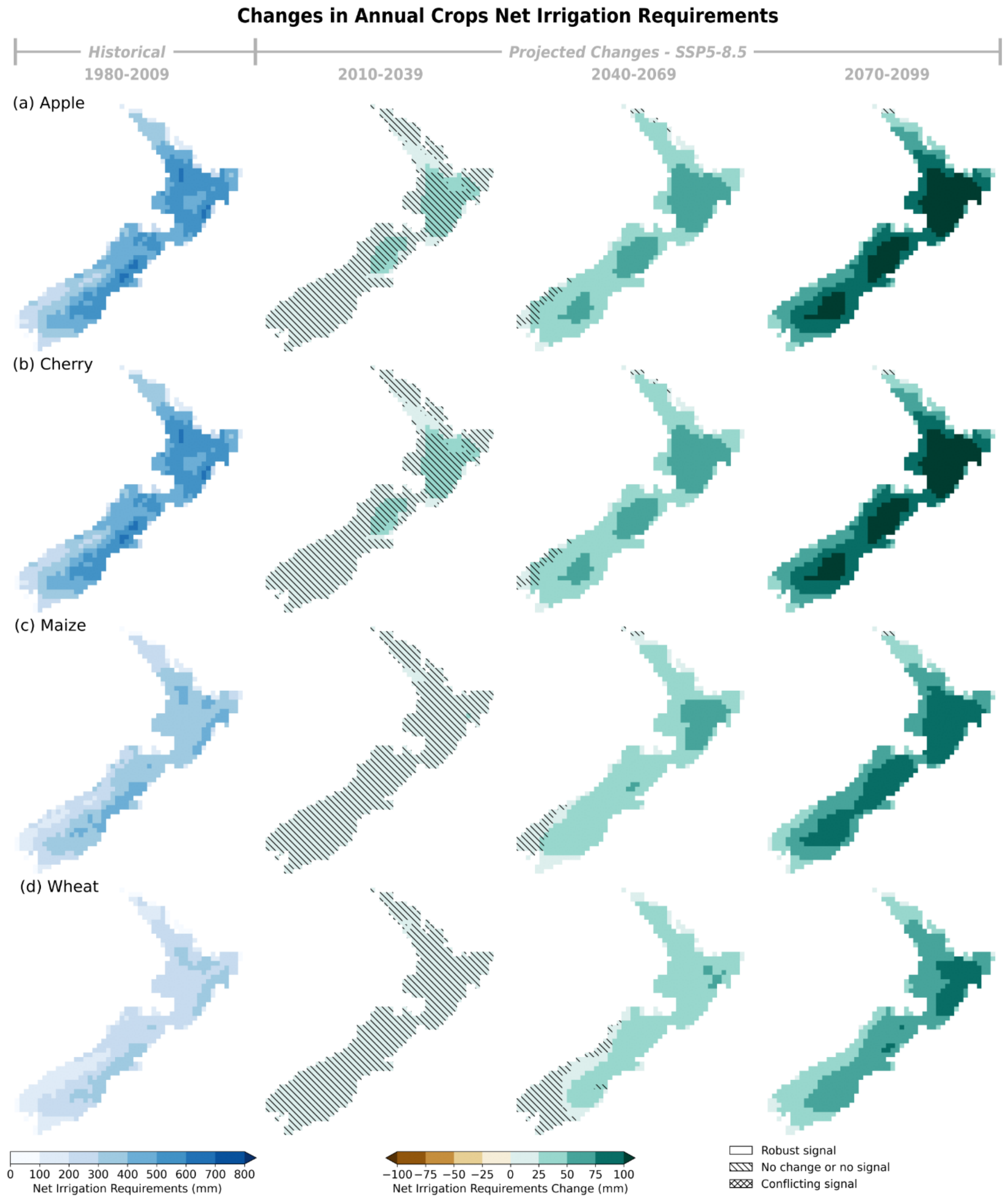

***Figure S11 | Historic annual crops net irrigation requirements (NIR) and near-, mid- and long-term changes**. Displayed are projected spatial patterns of multi-model (n=5) mean change in annual crop net irrigation requirements (NIR) with SSP5-8.5 in 2010-2039, 2040-2069 and 2070-2099 relative to the 1980-2009 historic NIR for **(a)** apple, **(b)** cherry, **(c)** maize and **(d)** wheat. The overlay represents the robustness[6]: no overlay indicates areas where the change is robust; diagonal lines indicate areas with no change or no robust significant change; crossed lines indicate areas of conflicting signals — maps generated in Python using matplotlib 3.9.2 (https://matplotlib.org).*

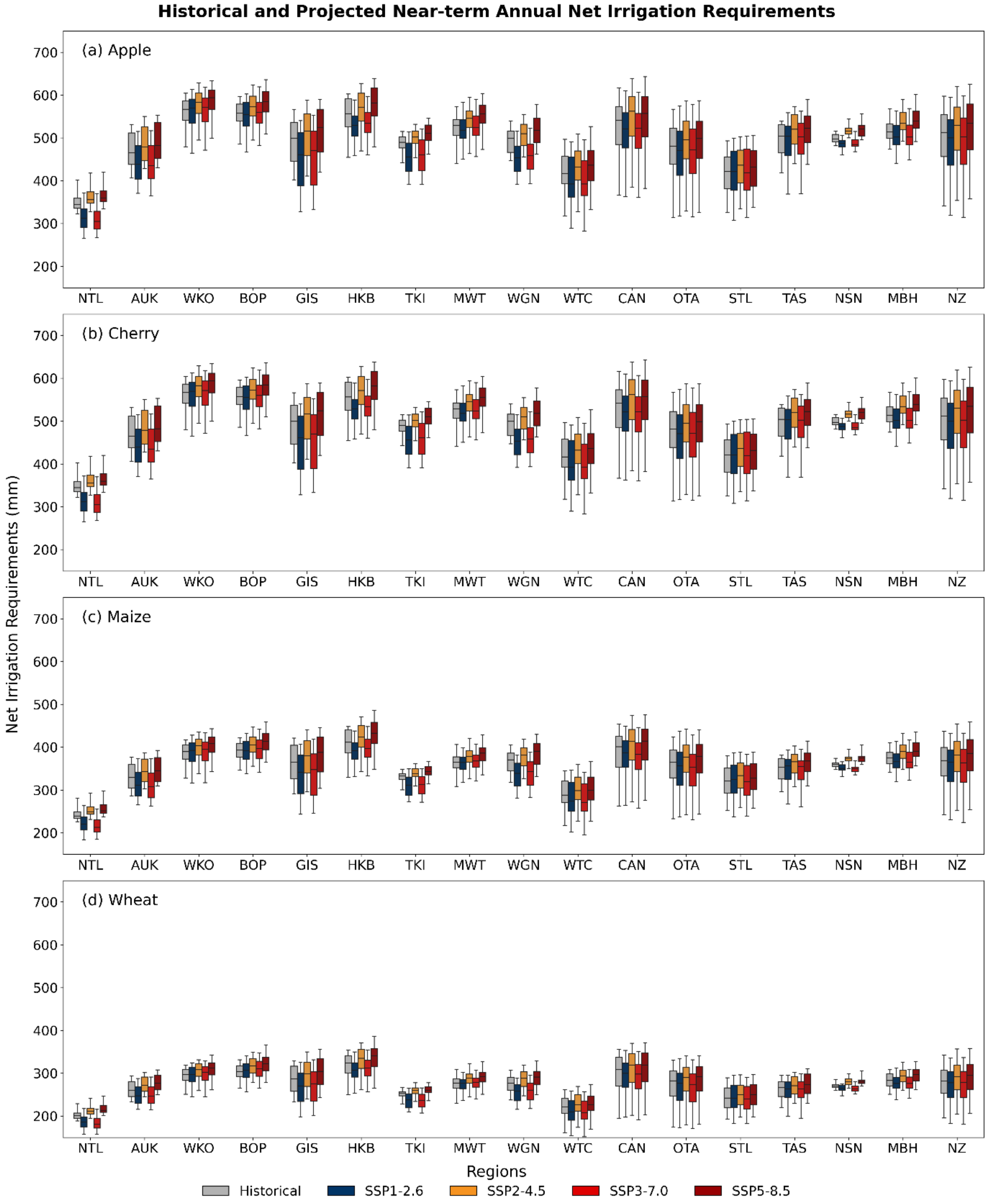

***Figure S12 | Historic and near-term annual crops net irrigation requirements (NIR)**. Presented are the historic (1980-2009) and near-term (2010-2039) multi-model (n=5) mean of the annual net irrigation requirements (NIR) for the SSP1-2.6, SSP2-4.5, SSP3-7.0 and SSP5-8.5, and **(a)** apple, **(b)** cherry, **(c)** maize and **(d)** wheat. The whiskers correspond to the multi-model mean interquartile range. The NIR are presented for each of the 16 New Zealand administrative regions and totals for New Zealand. Only the potential agricultural area was considered (see Spatial Statistical Analysis in Supplementary Methods). Regions: Northland (NTL), Auckland (AUK), Waikato (WKO), Bay of Plenty (BOP), Gisborne (GIS), Hawke's Bay (HKB), Taranaki (TKI), Manawatū-Whanganui (MWT), Wellington (WGN), West Coast (WTC), Canterbury (CAN), Otago (OTA), Southland (STL), Tasman (TAS), Nelson (NSN), Marlborough (MBH), New Zealand (NZ).*

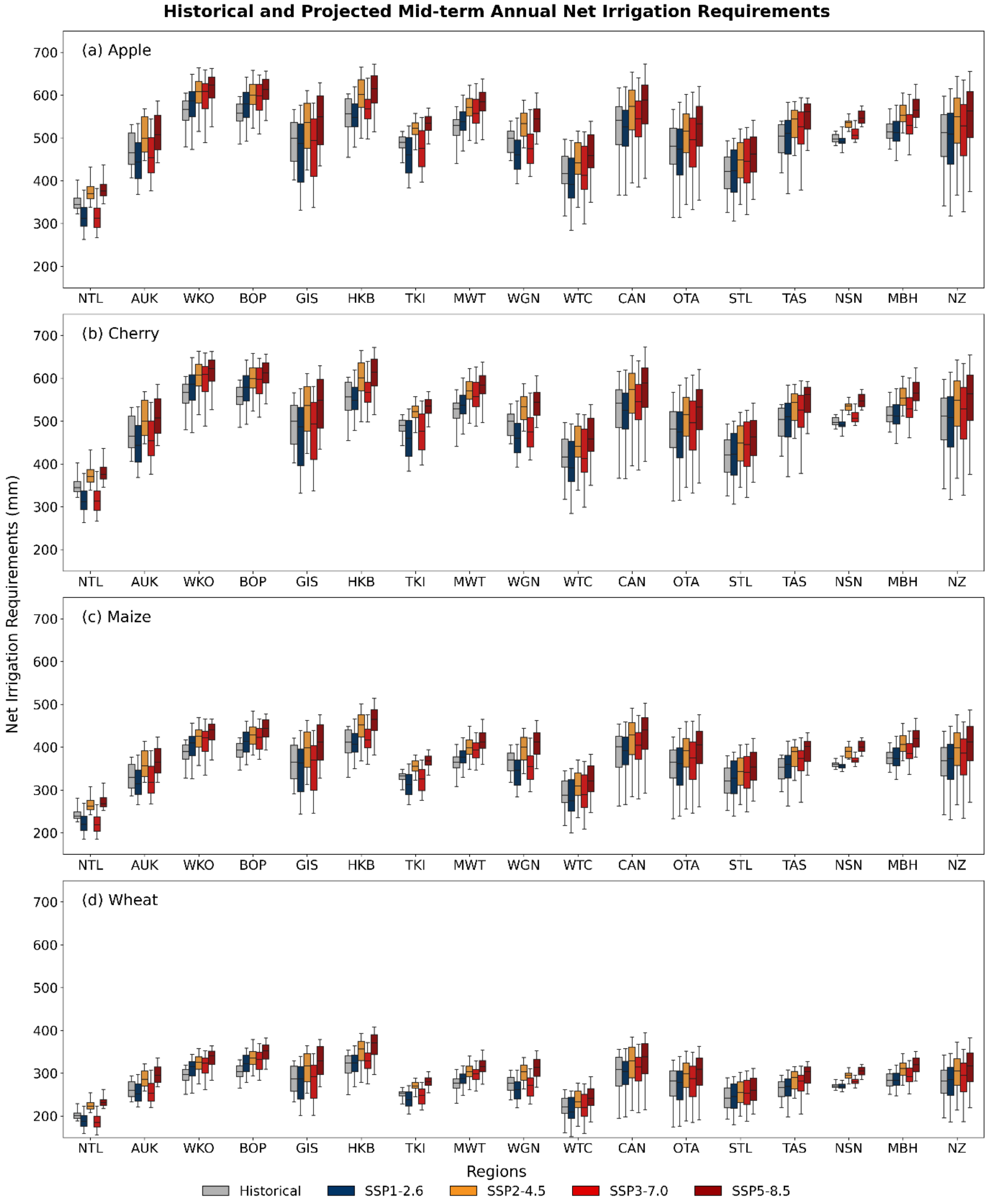

Figure S13 | Historic and mid-term annual crops net irrigation requirements (NIR)*. Presented are the historic (1980-2009) and mid-term (2040-2069) multi-model (n=5) mean of the annual net irrigation requirements (NIR) for the SSP1-2.6, SSP2-4.5, SSP3-7.0 and SSP5-8.5, and* ***(a)*** *apple,* ***(b)*** *cherry,* ***(c)*** *maize and* ***(d)*** *wheat. The whiskers correspond to the multi-model mean interquartile range. The NIR are presented for each of the 16 New Zealand administrative regions and totals for New Zealand. Only the potential agricultural area was considered (see Spatial Statistical Analysis in Supplementary Methods). Regions: Northland (NTL), Auckland (AUK), Waikato (WKO), Bay of Plenty (BOP), Gisborne (GIS), Hawke's Bay (HKB), Taranaki (TKI), Manawatū-Whanganui (MWT), Wellington (WGN), West Coast (WTC), Canterbury (CAN), Otago (OTA), Southland (STL), Tasman (TAS), Nelson (NSN), Marlborough (MBH), New Zealand (NZ).*

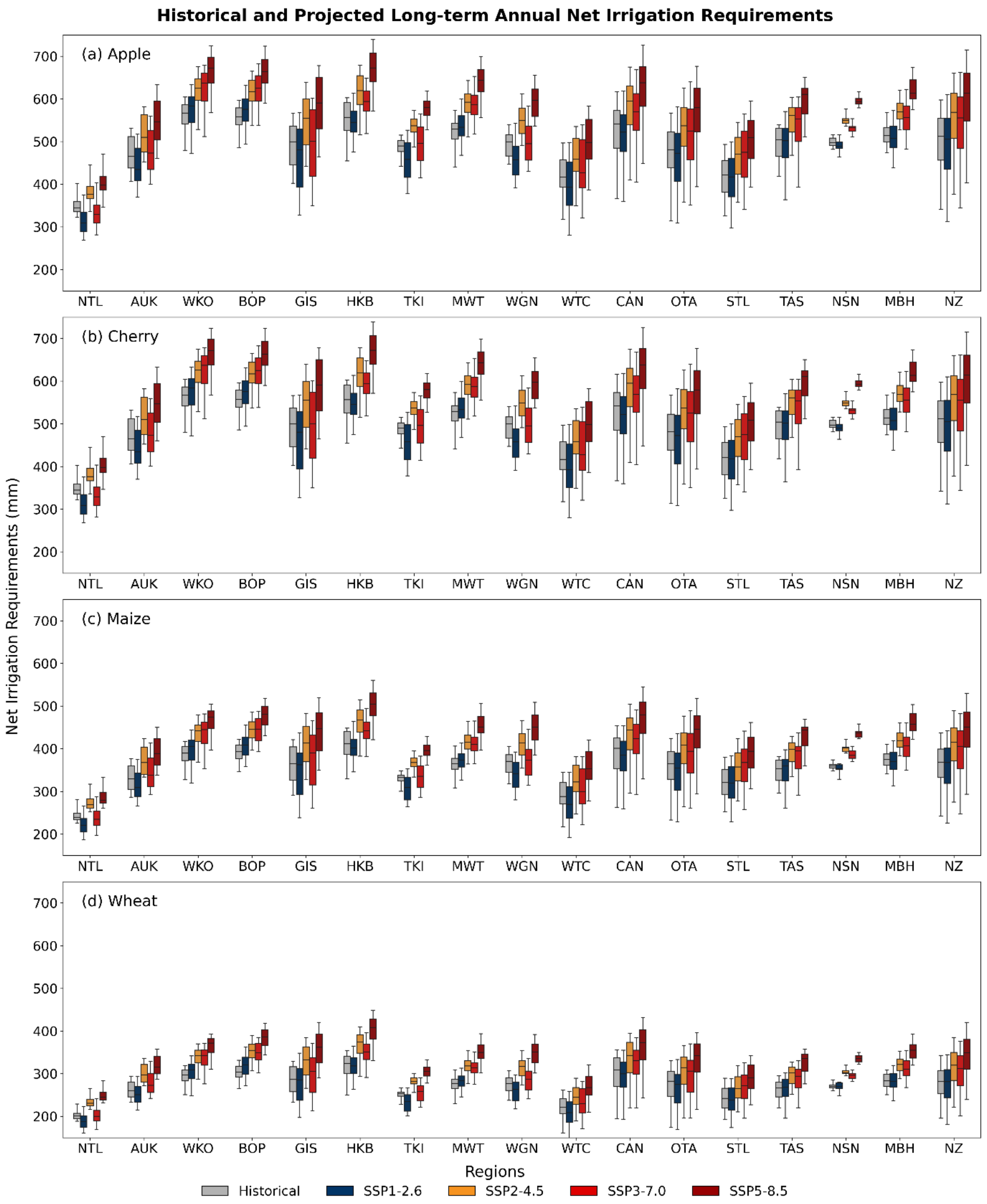

***Figure S14 | Historic and long-term annual crops net irrigation requirements (NIR)**. Presented are the historic (1980-2009) and long-term (2070-2099) multi-model (n=5) mean of net irrigation requirements (NIR) for the SSP1-2.6, SSP2-4.5, SSP3-7.0 and SSP5-8.5, and **(a)** apple, **(b)** cherry, **(c)** maize and **(d)** wheat. The whiskers correspond to the multi-model mean interquartile range. The NIR are presented for each of the 16 New Zealand administrative regions and totals for New Zealand. Only the potential agricultural area was considered (see Spatial Statistical Analysis in Supplementary Methods). Regions: Northland (NTL), Auckland (AUK), Waikato (WKO), Bay of Plenty (BOP), Gisborne (GIS), Hawke's Bay (HKB), Taranaki (TKI), Manawatū-Whanganui (MWT), Wellington (WGN), West Coast (WTC), Canterbury (CAN), Otago (OTA), Southland (STL), Tasman (TAS), Nelson (NSN), Marlborough (MBH), New Zealand (NZ).*

References

1. Vetharaniam, I. *et al*. Modelling Continuous Location Suitability Scores and Spatial Footprint of Apple and Kiwifruit in New Zealand. *Land* **11**, 1528 (2022).
2. Vetharaniam, I. et al. *Modelling the Effect of Climate Change on Land Suitability for Growing Perennial Crops. 362 (2021).*
3. *Thomas, S. M. et al. Evaluation of Profitability and Future Potential for Low Emission Productive Uses of Land That Is Currently Used for Livestock. (2020).*
4. *Thomas, S. M. et al. Exploring the role of high-value crops to reduce agricultural greenhouse gas emissions in New Zealand. Reg Environ Change 24, 105 (2024).*
5. *Allen, R. G., Pereira, L. S., Raes, D. & Smith, M. Crop Evapotranspiration: Guidelines for Computing Crop Water Requirements. (1998).*
6. *IPCC. Climate Change 2021: The Physical Science Basis. Contribution of Working Group I to the Sixth Assessment Report of the Intergovernmental Panel on Climate Change. (Cambridge University Press, Cambridge, UK and New York, NY, USA, 2021).*
